# Clec4A4 is a regulatory receptor for dendritic cells that impairs inflammation and T-cell immunity

**DOI:** 10.1038/ncomms11273

**Published:** 2016-04-12

**Authors:** Tomofumi Uto, Tomohiro Fukaya, Hideaki Takagi, Keiichi Arimura, Takeshi Nakamura, Naoya Kojima, Bernard Malissen, Katsuaki Sato

**Affiliations:** 1 Division of Immunology, Department of Infectious Diseases, Faculty of Medicine, University of Miyazaki, 5200 Kihara, Kiyotake, Miyazaki, 889-1692, Japan; 2 Department of Oral and Maxillofacial Surgery, Faculty of Medicine, University of Miyazaki, 5200 Kihara, Kiyotake, Miyazaki, 889-1692, Japan; 3 Department of Otolaryngology, Head and Neck Surgery, Faculty of Medicine, University of Miyazaki, 5200 Kihara, Kiyotake, Miyazaki, 889-1692, Japan; 4 Department of Applied Biochemistry, Tokai University, 4-1-1 Kita-kaname, Hiratsuka-shi, 259-1292 Kanagawa, Japan; 5 Centre d'Immunologie de Marseille-Luminy, Université de la Méditerrannée, Case 906, Institut National de la Santé et de la Recherche Médicale U631, and Centre National de la Recherche Scientifique UMR6102, Marseille 13288, France; 6 Japan Science and Technology Agency, Precursory Research for Embryonic Science and Technology (PRESTO), 4-1-8 Hon-cho, Kawaguchi, Saitama 332-0012, Japan

## Abstract

Dendritic cells (DCs) comprise several subsets that are critically involved in the initiation and regulation of immunity. Clec4A4/DC immunoreceptor 2 (DCIR2) is a C-type lectin receptor (CLR) exclusively expressed on CD8α^−^ conventional DCs (cDCs). However, how Clec4A4 controls immune responses through regulation of the function of CD8α^−^ cDCs remains unclear. Here we show that Clec4A4 is a regulatory receptor for the activation of CD8α^−^ cDCs that impairs inflammation and T-cell immunity. *Clec4a4*^−/−^CD8α^−^ cDCs show enhanced cytokine production and T-cell priming following Toll-like receptor (TLR)-mediated activation. Furthermore, *Clec4a4*^−/−^ mice exhibit TLR-mediated hyperinflammation. On antigenic immunization, *Clec4a4*^−/−^ mice show not only augmented T-cell responses but also progressive autoimmune pathogenesis. Conversely, *Clec4a4*^−/−^ mice exhibit resistance to microbial infection, accompanied by enhanced T-cell responses against microbes. Thus, our findings highlight roles of Clec4A4 in regulation of the function of CD8α^−^ cDCs for control of the magnitude and quality of immune response.

## Abstract (web summary)

Clec4A4 is a C-type lectin receptor highly expressed by CD8α^−^ dendritic cells. Here the authors show that its loss of function results in enhanced T cell responses and exacerbated autoimmunity, implicating Clec4A4 in limiting activation of the CD8α^−^ dendritic cells.

## Graphical abstract

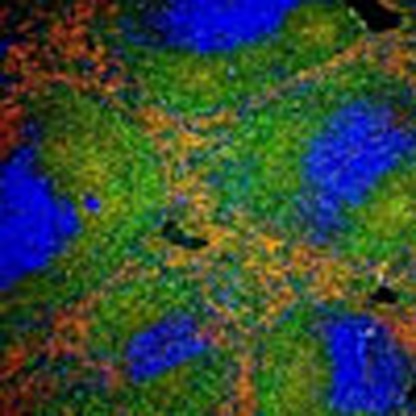

Dendritic cells (DCs) are essential antigen (Ag)-presenting cells (APCs) linking innate and adaptive immunity, which comprise heterogeneous subsets, functionally classified into conventional DCs (cDCs) and plasmacytoid DCs (pDCs)^1^,^2^,^3^. DCs act as sentinels to sense the invading pathogens through a variety pattern-recognition receptors (PRRs), including Toll-like receptors (TLRs).

Most of the members of TLRs, except for TLR3, associate myeloid factor88 (MyD88) on binding of the ligands, and initiates the activation of various intracellular signalling cascades, including interleukin (IL)-1 receptor-associated kinase 1/4 (IRAK1/4), tumour necrosis factor (TNF) receptor-associated factor 6 (TRAF6), and inhibitor of nuclear factor κB (NF-κB) kinase-β (IKK-β) and their downstream, which further activate NF-κB and mitogen-activated protein kinases (MAPKs), including ERK, JNK and p38, are involved in the secretion of proinflammatory cytokines, while the activation of IKK-α and its downstream interferon (IFN) regulatory factor (IRF)-7 are crucial for the production of type I IFN (IFN-I) (refs 4, 5, 6).

The recognition of the ligands by TLR3 or TLR4 also causes their recruitment of the adapters Toll/IL-1 receptor (IL-1R) domain-containing adapter-inducing interferon (IFN)-β (TRIF), which interacts with TANK-binding kinase 1 (TBK1) and activates, and downstream IRF-3 for the production of IFN-I, while TRIF also activates MAP kinases and NF-κB leading to the production of proinflammatory cytokines^4^,^5^.

DCs process and present exogenous Ags to CD4^+^ T cells through major histocompatibility complex class (MHC) II-restricted pathway for induction of CD4^+^ effector T (CD4^+^ T_eff_) cells^7^,^8^. DCs also prime CD8^+^ T cells mediated by classical MHC I presentation pathway of endogenously synthesized proteins and MHC I-dependent cross-presentation pathway of exogenous Ags for the generation of cytotoxic T lymphocytes (CTLs)^7^,^8^.

Mouse CD11c^+^ cDCs in lymphoid organs consist of two major subsets, classified into CD8α^+^ cDCs (cDC1) mainly residing in the T-cell zone and CD8α^−^ cDCs (cDC2) localizing in the red pulp and marginal zone, including CD4^+^CD8α^−^ cDCs and CD4^−^CD8α^−^ cDCs, which may exert distinct functions in immune responses^1^,^2^,^3^. CD8α^+^ DCs appear to be specialized for the phagocytosis of dead cells and the cross-presentation of cell-associated and soluble Ags to induce strong CTL responses^7^,^8^,^9^,^10^. In contrast, CD8α^−^ cDCs are more efficient than CD8α^+^ DCs in MHC II-dependent presentation pathway to initiate CD4^+^ T-cell responses^7^,^11^.

C-type lectin receptors (CLRs) have been known as PRRs recognizing a wide variety of glycan structures present on pathogens, commensals and self-glycoproteins using their carbohydrate recognition domain (CRD) in their extracellular portion, and the ligand binding of CLRs on the surface of myeloid cells often induces their internalization and/or intracellular signalling events^12^,^13^. Several CLRs act as activation receptors that transduce intracellular signals via an integral immunoreceptor tyrosine-based activation motif (ITAM)-like motif within their cytoplasmic tails, or via interaction with ITAM-bearing Fc receptor (FcR)γ adaptor molecules^13^,^14^. Other CLRs possess an immunoreceptor tyrosine-based inhibition motif (ITIM) in their cytoplasmic portions, and intracellular signalling from these CLRs suppresses or paradoxically enhances cellular activation^15^,^16^,^17^.

DC subsets reportedly express unique repertoires of CLRs that are involved in Ag internalization and the routing to MHC I and II loading compartments, thereby influencing T-cell responses^7^,^18^. In a steady state, DEC-205 and Clec9A are predominantly expressed on CD8α^+^ DCs^7^,^18^. In contrast, CD8α^−^ cDCs specifically express Clec4A4 (DCIR2) recently found to be recognized by the 33D1 monoclonal antibody (mAb), which has been widely utilized as a benchmark for the detection of mouse cDCs^7^,^19^,^20^. Clec4A4 has the potential to deliver negative signals due to the presence of an ITIM in its cytoplasmic portion that possibly affects the cellular activation of CD8α^−^ cDCs. However, the physiological role for Clec4A4 in the regulation of immune responses through control of the function of CD8α^−^ cDCs remains unclear because its function is poorly characterized relative to those of other CLRs.

In this study, we show by characterizing Clec4A4-deficient mice that Clec4A4 acts as a unique regulatory CLR in the context of TLR-mediated activation of CD8α^−^ cDCs. We further demonstrate that DCIR2 plays a crucial role in the regulation of innate and adaptive immune responses under inflammatory conditions *in vivo*. Collectively, these findings indicate that Clec4A4 provides fine tuning of the function of major cDC subsets to generate appropriate immune responses.

## Results

### Clec4A4 suppresses the function of cDCs

**Figure 1:**
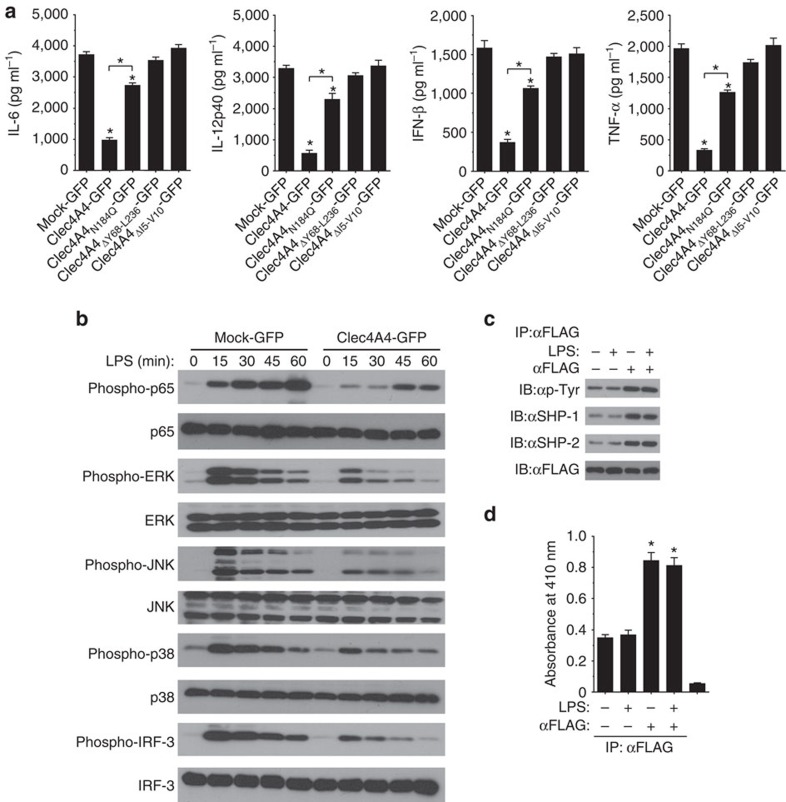
Retroviral transduction of Clec4A4 suppresses the TLR-mediated activation of cDCs. (**a**) BMDCs expressing mock-GFP, Clec4A4-GFP, Clec4A4_N186Q_-GFP, Clec4A4_ΔY68–L236_-GFP or Clec4A4_ΔI5–V10_-GFP were stimulated or not stimulated with the indicated LPS, and the production of cytokines was measured by enzyme-linked immunosorbent assay (ELISA). Data are the mean±s.d. from three individual samples in a single experiment. *P<0.01 compared with BMDCs expressing mock-GFP or among groups (analysis of variance (ANOVA), Bonferroni's multiple comparison test). (**b**) BMDCs expressing mock-GFP or Clec4A4-GFP were stimulated or not stimulated with LPS for the period indicated, at which time cells were lysed. Total lysate was analysed using Ab specific for p65, ERK, JNK, p38 and IRF-3 or for phosphorylated versions of these proteins. (**c**,**d**) BMDCs expressing mock-GFP or Clec4A4-GFP were stimulated or not stimulated with LPS in combination with or without crosslinking of Clec4A4 with anti-FLAG M2 mAb for 30 min. (**c**) The immunoprecipitate with anti-FLAG M2 mAb was analysed using anti-phosphotyrosine (p-Tyr) mAb, anti-SHP-1 mAb, anti-SHP-2 mAb or anti-FLAG M5 mAb. (**d**) The immunoprecipitate with anti-FLAG M2 mAb or control IgG was analysed for protein tyrosine phosphatase activity. Data are the mean±s.d. from three individual samples in a single experiment. *P<0.01 compared with the immunoprecipitate with anti-FLAG M2 mAb obtained from unstimuated cells (ANOVA, Bonferroni's multiple comparison test). All data are representative of at least three independent experiments.

To address the function of Clec4A4 in cDCs, bone marrow (BM)-derived DCs (BMDCs) as well as cDC cell lines (DC2.4) (ref. 21), which did not express of Clec4A4 on the cell surface (Supplementary Fig. 1a,b), were transfected with a bicistronic retroviral vector carrying an internal ribosome entry site followed by green fluorescent protein (IRES-GFP; mock-GFP) or C terminus FLAG-tagged Clec4A4-IRES-GFP (Clec4A4-GFP) (Supplementary Fig. 1a,b), and their production of cytokines was analysed after stimulation with various TLR ligands. Following stimulation with Pam3CSK4 (TLR1/TLR2 ligands), poly(I:C) (TLR3 ligand), lipopolysaccharide (LPS; TLR4 ligand) or CpG-B (TLR9 ligand), BMDCs or DC2.4-expressing Clec4A4-GFP showed a lower production of IL-6 IL-12p40, IFN-β and TNF-α than BMDCs or DC2.4-expressing mock-GFP (Fig. 1a; Supplementary Fig. 1c,d; Supplementary Fig. 2a).

To address how Clec4A4 regulates the TLR-mediated cytokine production in cDCs, we examined the signalling mechanism by which the expression of Clec4A4 caused the reduced production of cytokines in cDCs. BMDCs or DC2.4-expressing Clec4A4-GFP showed lower phosphorylation of NF-κB p65 subunit, ERK, JNK and p38 as well as IRF-3 or IRF-7 than BMDCs or DC2.4-expressing mock-GFP after stimulation with LPS or CpG-B (Fig. 1b; Supplementary Fig. 1e; Supplementary Fig. 2b).

We also investigated the activation status of the ITIM in Clec4A4 after the ligations of TLRs and Clec4A4. The steady-state phosphorylation of the ITIM in Clec4A4 as well as its association with SHP-1 and SHP-2 was observed in BMDCs expressing Clec4A4-GFP, while crosslinking of Clec4A4 with anti-FLAG mAb, but not stimulation with LPS, enhanced its phosphorylation status and recruitment of these phosphatases (Fig. 1c). We also observed that tyrosine-specific phosphatase activity in the immunoprecipitate with Clec4A4 was further enhanced following crosslinking of Clec4A4 with anti-FLAG mAb (Fig. 1d).

Clec4A4 consists of 236 amino acid, in which Y68 to L236 or I5 to V10 correspond to the extracellular portion or the ITIM sequence, respectively. To clarify the role of extracellular portion or ITIM in the suppressive function of Clec4A4 on the activation of cDCs, we generated BMDCs expressing Clec4A4 mutant lacking extracellular portion (Clec4A4_ΔY68–L236_-GFP) or ITIM in cytoplasmic tails (Clec4A4_ΔI5–V10_-GFP). Following stimulation with LPS or CpG-B, there was no marked difference in the TLR-mediated cytokine production among BMDCs expressing mock-GFP, BMDCs expressing Clec4A4_ΔY68–L236_-GFP, and BMDCs expressing Clec4A4_ΔI5–V10_-GFP (Fig. 1a; Supplementary Fig. 2a), indicating that the inhibitory effect of Clec4A4 on the TLR-mediated activation of cDCs requires both extracellular portion and ITIM in cytoplasmic tails.

Collectively, these results indicate that retroviral transfection of Clec4A4 suppresses the TLR-mediated cytokine production mediated through the impaired activation of the downstream signalling cascades in cDCs.

### CRD–glycan interactions mediate homotypic Clec4A4 binding

**Figure 2:**
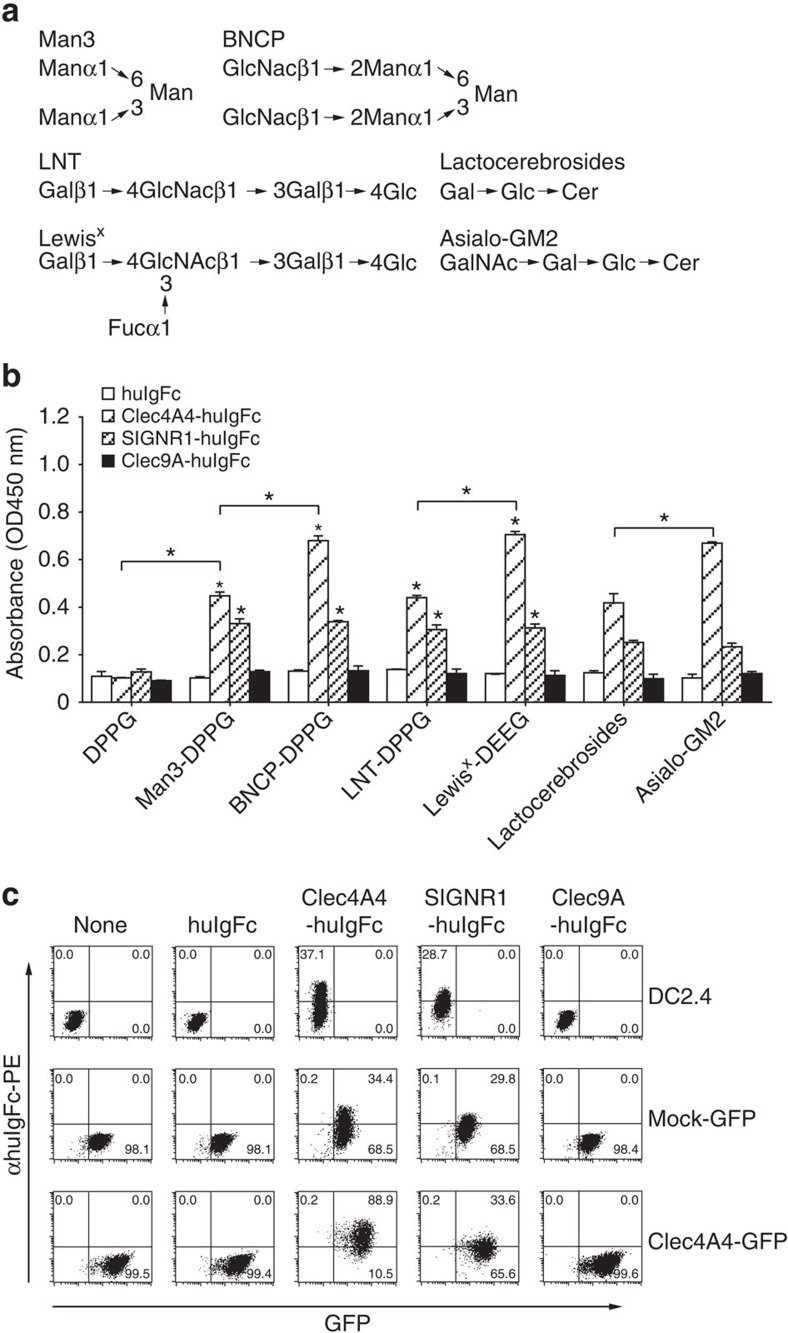
Self-interaction of Clec4A4 through the binding of CRD and glycans. (**a**) Schematic diagram of structures of the oligosaccharides in NGL. Gal, D-galactose; Glc, D-glucose. (**b**) The binding of huIgFc, Clec4A4-huIgFc, SIGNR1-huIgFc or Clec9A-huIgFc to glycans was measured by ELISA. Data are the mean±s.d. from three individual samples in a single experiment. *P<0.01 compared with DEEG or among groups (analysis of variance (ANOVA), Bonferroni's multiple comparison test). (**c**) The binding of huIgFc, Clec4A4-huIgFc, SIGNR1-huIgFc or Clec9A-huIgFc to DC2.4, DC2.4-expressing mock-GFP, and DC2.4-expressing Clec4A4-GFP was analysed by flow cytometry with anti-huIgFc-PE. Data are presented by a dot plot, and numbers represent the proportion of the binding of huIgFc, Clec4A4-huIgFc, SIGNR1-huIgFc or Clec9A-huIgFc in each quadrant. All data are representative of at least three independent experiments.

To identify the ligands of Clec4A4, we created CLR fusion protein, which consists of the extracellular domain of Clec4A4 and the Fc fragment of human immunoglobulin G (IgG) (Clec4A4-huIgFc). As the CRD of the family of Clec4As potentially recognizes oligosaccharide moieties^17^,^22^, we examined whether Clec4A4-huIgFc would bind to neoglycolipids (NGL) constructed with oligosaccharides and dipalmitoylphosphatidylglycerol (DPPG)^23^ as carrier lipid (Fig. 2a,b). Clec4A4-huIgFc, but not huIgFc and Clec9A-huIgFc, exhibited a more potent specific binding to mannotriose (Man3)-DPPG or lacto-*N*-fucopentaose 3 (LNFP3 (Lewis^x^))-DPPG than DPPE alone or lacto-*N*-tetraose (LNT)-DPPG. On the other hand, the specific binding of Clec4A4-huIgFc, but not huIgFc and Clec9A-huIgFc, to biantennary *N*-linked core pentasaccharide (BNCP)-DPPG, which contains *N*-acetylglucosamine (GlcNAc) and Man3 residues, was higher than Man3-DPPG. We also observed that Clec4A4-huIgFc, but not huIgFc and Clec9A-huIgFc, specifically bound to asialoganglioside-GM2 (asialo-GM2) consisting of *N*-acetylgalactosamine (GalNAc) and lactocerebrosides was higher than that to lactocerebrosides. As the CRD of SIGNR1 also reportedly recognizes several oligosaccharide residues of glycan^23^, SIGNR1-huIgFc showed higher specific binding to Man3-DPPG than DPPG alone, whereas the binding of SIGNR1-huIgFc with BNCP-DPPG or Lewis^x^-DPPG was comparable to that with Man3-DPPG or LNT-DPPG. Furthermore, there was no different in the binding of SIGNR1-huIgFc to lactocerebrosides and asialo-GM2.

Taken together, these results indicate that the CRD of Clec4A4 specifically recognizes D-mannose (Man), L-fucose (Fuc), GlcNAc and GalNAc moieties on glycan.

We further examined whether Clec4A4-huIgFc directly bound to DC2.4-expressing Clec4A4-GFP because the extracellular domain of Clec4A4 possesses several predicted *N*-glycosylation sites (Fig. 2c). While Clec4A4-huIgFc, but not huIgFc and Clec9A-huIgFc, bound to DC2.4 and DC2.4-expressing mock-GFP, the specific binding of Clec4A4-huIgFc to DC2.4-expressing Clec4A4-GFP was significantly higher than that to DC2.4 and DC2.4-expressing mock-GFP, indicating that Clec4A4 interacts with itself on cDCs. To a lesser degree, similar results were observed in the binding of SIGNR1-huIgFc to DC2.4, DC2.4-expressing mock-GFP and DC2.4-expressing Clec4A4-GFP.

Since the self-interaction of Clec4A4 would be mediated by the binding of the CRD with oligosaccharide resides on glycans, and the N186 of Clec4A4 is a only *N*-glycosylation site in the CRD (ref. 17), we therefore generated BMDCs expressing a glycosylation mutant of Clec4A4 (Clec4A4_N186Q_-GFP) lacking the *N*-glycosylation site in the CRD. The introduction of Clec4A4_N186Q_-GFP displayed a lower inhibitory effect on the capacity of BMDCs to secrete cytokines after stimulation with LPS or CpG-B as compared with that of Clec4A4-GFP (Fig. 1a; Supplementary Fig. 2a), suggesting that the *N*-glycosylation of CRD is required for self-interaction of Clec4A4 for the suppressive effect on the TLR-mediated activation of cDCs.

### Clec4A4 inhibits the TLR-mediated activation of CD8α^−^ cDCs

**Figure 3:**
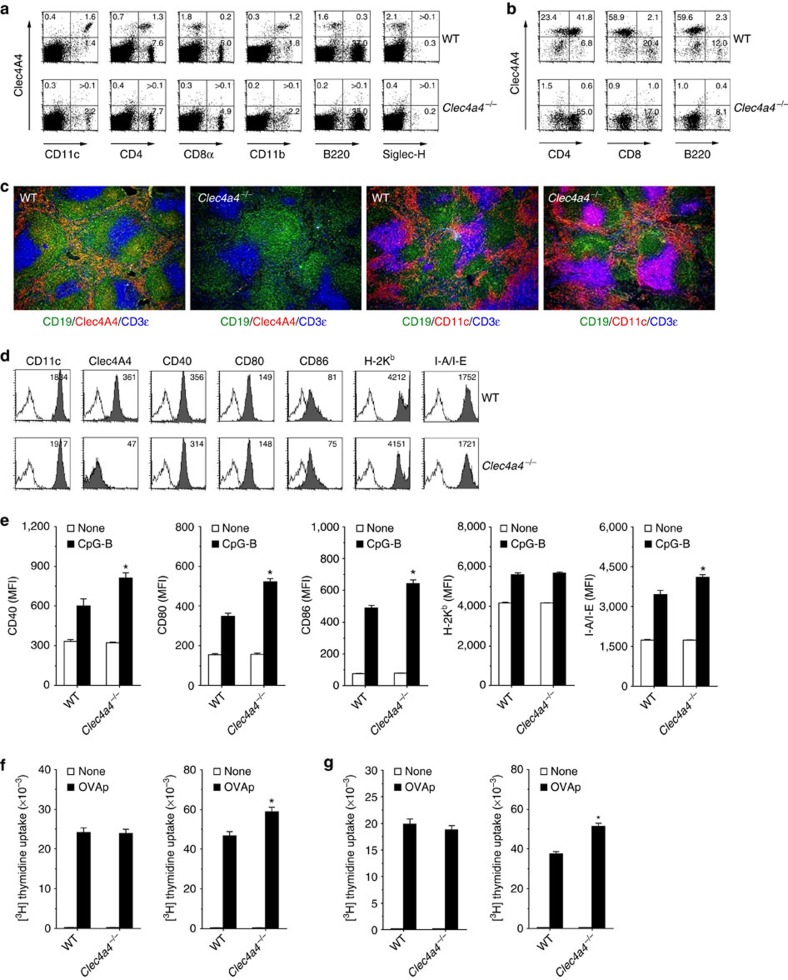
Deficiency of Clec4A4 modulates TLR-mediated activation of CD8α^−^ cDCs. (**a**,**b**) The expression of Clec4A4 and cell surface molecules on splenocytes (**a**) and CD11c^+^ DCs (**b**) obtained from WT mice and *Clec4a4*^−/−^ mice was analysed by flow cytometry. Data are presented by a dot plot, and numbers represent the proportion in each quadrant. (**c**) Immunofluorescent microscopic analysis was performed on frozen horizontal sections. Sections were stained for CD19 (green), CD3ɛ (blue) and Clec4A4 (red) or CD11c (red). (**d**) The expression of Clec4A4 and cell-surface molecules on CD8α^−^ cDCs obtained from WT mice and *Clec4a4*^−/−^ mice was analysed by flow cytometry. Data are presented by a histogram, and numbers represent mean fluorescence intensity (MFI). (**e**) WT mice (*n*=6) and *Clec4a4*^−/−^ mice (*n*=6) were injected with or without CpG-B, and CD8α^−^ cDCs were obtained 24 h after injection. The expression of cell surface molecules on CD8α^−^ cDCs was analysed by flow cytometry. Data are MFI±s.d. from six individual samples in a single experiment. (**f**,**g**) WT mice (*n*=6) and *Clec4a4*^−/−^ mice (*n*=6) were not injected (left panel) or injected (right panel) with CpG-B, and CD8α^−^ cDCs were obtained 24 h after injection. CD45.1^+^OT-II CD4^+^ T cells (**f**) or CD45.1^+^OT-I CD8^+^ T cells (**g**) were cultured with CD8α^−^ cDCs (10^4^) obtained from WT mice and *Clec4a4*^−/−^ mice in the presence or absence of OVA_323–339_ peptide (1 μM; **f**) or OVA_257–264_ peptide (1 nM; **g**), and the proliferation was measured by [^3^H]thymidine incorporation. Data are the mean±s.d. from six individual samples in a single experiment. *P<0.01 compared with WT mice (analysis of variance (ANOVA), Bonferroni's multiple comparison test). All data are representative of at least three independent experiments.

To investigate the physiological functions of Clec4A4 in the control of the immune responses through regulation of the function of CD8α^−^ cDCs, we created *Clec4a4*^−/−^ mice (Supplementary Fig. 3a–e), which were viable and healthy. As expected, CD8α^−^ cDCs from *Clec4a4*^−/−^ mice lacked cell surface expression of Clec4A4 (Fig. 3a,b). Histological analysis of Spl obtained from *Clec4a4*^−/−^ mice confirmed that the expression of Clec4A4 was not detected, while CD11c^+^ DCs were normally localized (Fig. 3c). CD8α^−^ cDCs obtained from wild-type (WT) mice and *Clec4a4*^−/−^ mice had similar expressions of MHC I (H-2K^b^) and MHC II (I-A/I-E) as well as CD11c and costimulatory molecules (Fig. 3d,e) under steady-state conditions. We also observed similar ability of CD8α^−^ cDCs between WT mice and *Clec4a4*^−/−^ mice to activate CD4^+^ and CD8^+^ T cells using ovalbumin (OVA)-specific T-cell receptor (TCR) transgenic OT-II CD4^+^ T cells and OT-I CD8^+^ T cells (refs 6, 24; Fig. 3f,g).

To address the influence of deficiency of Clec4A4 on the functions of CD8α^−^ cDCs, we analysed the TLR-mediated changes of CD8α^−^ cDCs from WT mice and *Clec4a4*^−/−^ mice. Whereas the injection of CpG-B enhanced the expression of MHC I, MHC II, and costimulatory molecules on CD8α^−^ cDCs, and their ability to activate OT-II CD4^+^ T cells and OT-I CD8^+^ T cells in WT mice, compared with those from untreated WT mice, the expression levels of these molecules and their ability to activate T cells were further enhanced in *Clec4a4*^−/−^ mice (Fig. 3e–g; Supplementary Fig. 4).

Collectively, the deficiency of Clec4A4 promotes the TLR-mediated upregulation of MHC and costimulatory molecules in CD8α^−^ cDCs, leading to their enhancement of T-cell activation.

**Figure 4:**
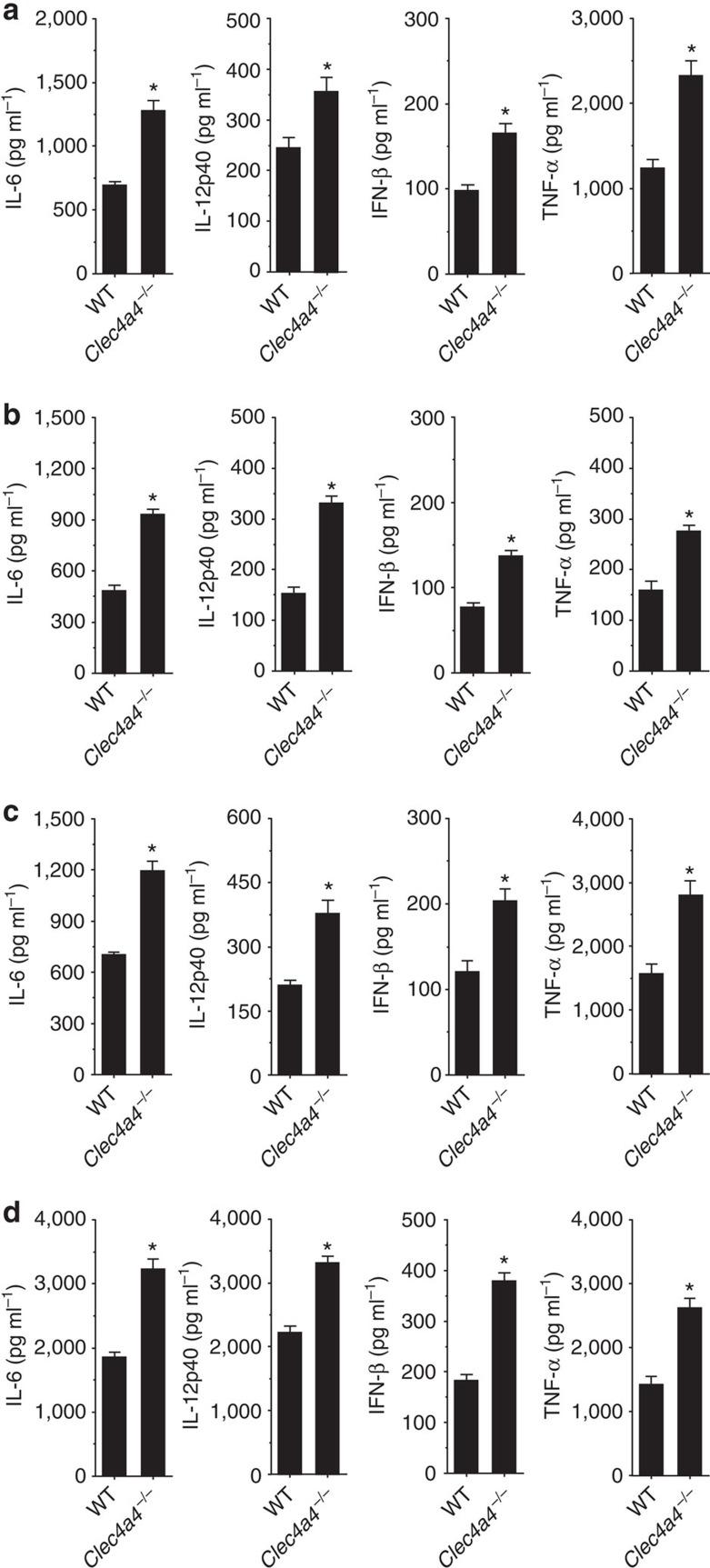
Deficiency of Clec4A4 enhances the ability of CD8α^−^ cDCs to produce cytokines in response to TLR ligands. CD8α^−^ cDCs obtained from WT mice and *Clec4a4*^−/−^ mice were stimulated or not stimulated with Pam3CSK4 (**a**), poly(I:C) (**b**), LPS (**c**) and CpG-B (**d**), and the production of cytokines was measured by ELISA. Data are the mean±s.d. from three individual samples in a single experiment. *P<0.01 compared with WT mice (analysis of variance (ANOVA), Bonferroni's multiple comparison test). All data are representative of at least three independent experiments.

Because the induced expression of Clec4A4 suppresses TLR-mediated cytokine production in cDCs, we compared the cytokine production of CD8α^−^ cDCs between WT mice and *Clec4a4*^−/−^ mice (Fig. 4). *Clec4a4*^−/−^ CD8α^−^ cDCs showed higher secretion of IL-6, IL-12p40, IFN-β and TNF-α after stimulation with Pam3CSK4, poly(I:C), LPS and CpG-B than WT CD8α^−^ cDCs. We also observed that *Clec4a4*^−/−^ CD8α^−^ cDCs showed greater phosphorylation of NF-κB p65 subunit, ERK, JNK and p38 as well as IRF-3 or IRF-7 after stimulation with LPS or CpG-B (Supplementary Fig. 5).

These results indicate that the deficiency of Clec4A4 enhances the ability of CD8α^−^ cDCs to produce proinflammatory cytokines and type I IFN, which is associated with the increased activation of NF-κB, MAPKs and IRFs in response to TLR ligands.

### Clec4A4 suppresses the TLR-mediated inflammation

**Figure 5:**
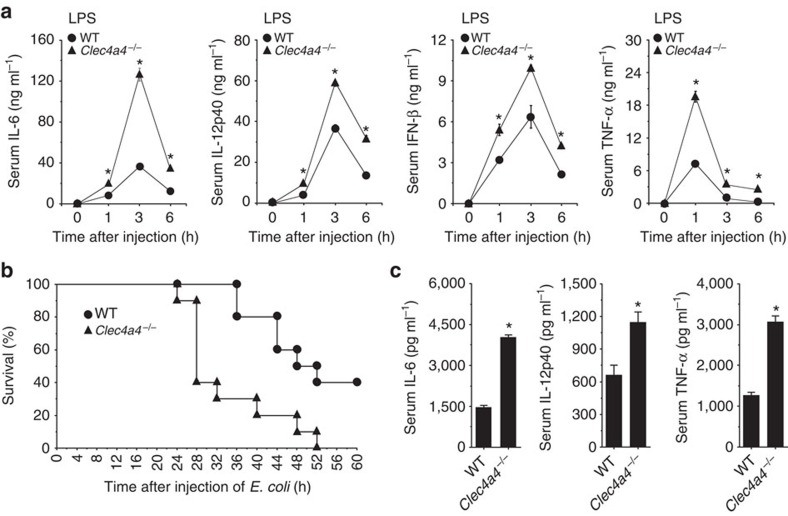
Deficiency of Clec4A4 leads to TLR-mediated hyperinflammatory response *in vivo*. (**a**) WT mice (*n*=6) and *Clec4a4*^−/−^ mice (*n*=6) were injected with LPS, and serum production of cytokines was measured at the indicated time after injection by ELISA. Data are the mean±s.d. from six individual samples in a single experiment. *P<0.01 compared with WT mice (analysis of variance (ANOVA), Bonferroni's multiple comparison test). (**b**,**c**) WT mice (*n*=10) and *Clec4a4*^−/−^ mice (*n*=10) were injected with heat-killed *E. coli*. (**b**) Survival rate was monitored at the indicated times for 60 h after injection of heat-killed *E. coli*. *P<0.01 compared with WT mice (Kaplan–Meier log-rank test). (**c**) Serum production of cytokines was measured 24 h after injection with heat-killed *E. coli* by ELISA. Data are the mean±s.d. from 10 individual samples in a single experiment. *P<0.01 compared with WT mice (analysis of variance (ANOVA), Bonferroni's multiple comparison test). All data are representative of at least three independent experiments.

We examined the roles of Clec4A4 in the TLR-mediated inflammation *in vivo*. The application of Pam3CSK4, poly(I:C), LPS or CpG-B caused serum secretion of IL-6, IL-12p40, IFN-β and TNF-α in WT mice (Fig. 5a and Supplementary Fig. 6). On the other hand, *Clec4a4*^−/−^ mice exhibited prominent elevation in the serum production of these cytokines following injection with various TLR ligands, compared with WT mice (Fig. 5a; Supplementary Fig. 6). We also demonstrated that *Clec4a4*^−/−^ mice showed more susceptiblity to lethality induced by microbial peritonitis than WT mice, and this lethality was correlated with the more marked secretion of serum proinflammatory cytokines 24 h following the administration of heat-killed *Escherichia coli* (Fig. 5b,c).

We next compared the proportion of leukocytes in the spleen between WT mice and *Clec4a4*^−/−^ mice under steady-state and TLR ligand-induced inflammatory conditions (Supplementary Fig. 7). In the steady-state conditions, *Clec4a4*^−/−^ mice exhibited regular myeloid and lymphoid immune cell compartments in the spleen. The administration of CpG-B to WT mice increased the weight of spleen and the absolute number of leukocytes, with elevated frequencies of CD11c^−^B220^+^ B cells and CD11c^+^B220^+^ pDCs and a reduced proportion of other leukocytes. On the other hand, *Clec4a4*^−/−^ mice showed greater weight of spleen and more splenocytes than WT mice after injection with CpG-B. Furthermore, *Clec4a4*^−/−^ mice exhibited a higher frequency of CD11c^−^B220^+^ B cells, and lower frequencies of CD3^+^ T cells, CD4^+^ T cells and CD8^+^ T cells than WT mice following injection with CpG-B.

Taken together, these results indicate that the deficiency of Clec4A4 augments TLR-mediated inflammation *in vivo*.

### Clec4a4 attenuates the responses of CD4^+^ T cells

**Figure 6:**
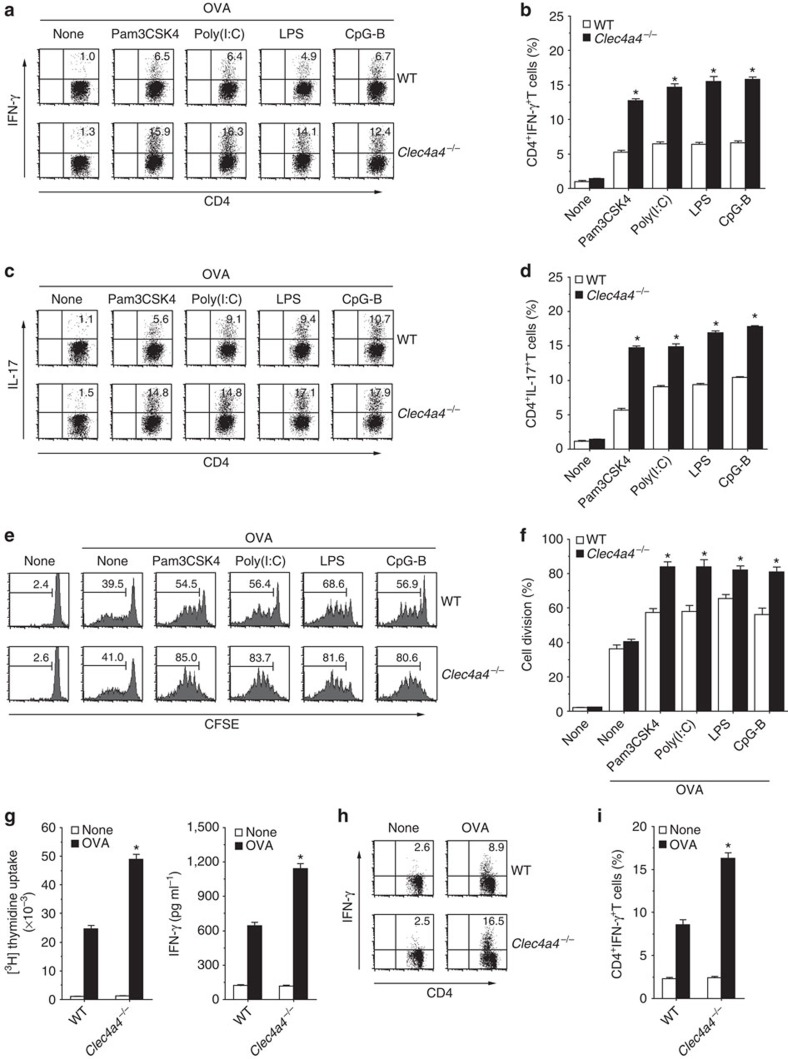
Deficiency of Clec4A4 increases Ag-specific CD4^+^ T-cell responses *in vivo*. (**a**–**d**) CD45.1^+^OT-II CD4^+^ T cells were cultured with CD8α^−^ cDCs obtained from WT mice and *Clec4a4*^−/−^ mice in the presence or absence of Pam3CSK4 (1 μg ml^−1^), poly(I:C) (50 μg ml^−1^), LPS (1 μg ml^−1^) or CpG-B (0.1 μM) in combination with OVA_323–339_ peptide under T_H_1 (**a**,**b**)- or T_H_17 (**c**,**d**)-polarized culture conditions for 3 days, and intracellular production of IFN-γ (**a**,**b**) or IL-17 (**c**,**d**) in the cultured CD4^+^ T cells was analysed by flow cytometry. (**a**,**c**) Data are presented by a dot plot, and numbers represent the proportion of IFN-γ^+^ cells (**a**) and IL-17^+^ cells (**c**) among gated CD4^+^ T cells in each quadrant. (**b**,**d**) Data are the mean percentage of positive cells±s.d. from three individual samples in a single experiment. (**e**,**f**) CFSE-labelled CD45.1^+^OT-II CD4^+^ T cells were transferred into WT mice (*n*=6) and *Clec4a4*^−/−^ mice (*n*=6), and then the mice were immunized with OVA protein in combination with or without the indicated TLR ligands. Ag-specific division of CD45.1^+^OT-II CD4^+^ T cells was analysed 3 days after the immunization by flow cytometry. (**e**) Data are presented by a histogram, and numbers represent the proportion of CFSE dilution among gated CD45.1^+^OT-II CD4^+^ T cells in each histogram. (**f**) Data are the mean percentage of positive cells±s.d. from six individual samples in a single experiment. (**g**–**i**) WT mice (*n*=6) and *Clec4a4*^−/−^ mice (*n*=6) were immunized with CFA plus OVA protein. At 14 days after the immunization, spleen CD4^+^ T cells were isolated then cultured with WT CD11c^+^ DCs in the presence or absence of OVA protein for the measurement of proliferative responses by [^3^H]thymidine incorporation (**g**, left panel) and production of IFN-γ (**g**, right panel) by ELISA. Data are the mean±s.d. from six individual samples in a single experiment. (**h**,**i**) Intracellular production of IFN-γ in the cultured CD4^+^ T cells was analysed by flow cytometry. (**h**) Data are presented by a dot plot, and numbers represent the proportion of IFN-γ^+^ cells among gated CD4^+^ T cells in each quadrant. (**i**) Data are the mean percentage of positive cells±s.d. from six individual samples in a single experiment. *P<0.01 compared with WT mice (analysis of variance (ANOVA), Bonferroni's multiple comparison test). All data are representative of at least three independent experiments.

We examined how Clec4A4 controls CD4^+^ T-cell responses through the regulation of the function of CD8α^−^ cDCs. *In vitro* analysis revealed that *Clec4a4*^−/−^ CD8α^−^ cDCs displayed an enhanced capacity to generate OVA-specific IFN-γ-producing CD4^+^ T cells (T helper 1; T_H_1 cells) and IL-17-producing CD4^+^ T cells (T_H_17 cells) when compared with WT CD8α^−^ cDCs (Fig. 6a–d; Supplementary Fig. 8a,b).

To determine the roles of Clec4A4 in the capacity of CD8α^−^ cDCs to prime Ag-specific CD4^+^ T cells *in vivo*, OT-II CD4^+^ T cells, which were labelled with carboxyfluorescein diacetate-succinimidyl ester (CFSE), were adoptively transferred into mice, and their division was monitored 3 days after administration of soluble OVA protein under steady-state or inflammatory conditions (Fig. 6e,f; Supplementary Fig. 8c). While similar Ag-specific division of OT-II CD4^+^ T cells between WT mice and *Clec4a4*^−/−^ mice was observed after systemic injection of OVA protein, *Clec4a4*^−/−^ mice exhibited the enhancement of this response compared with WT mice following the administration of OVA protein plus various TLR ligands.

We also compared the responses of Ag-specific CD4^+^ T cells between WT mice and *Clec4a4*^−/−^ mice. At 14 days after immunization with OVA protein and complete Freund's adjuvant (CFA), CD4^+^ T cells from *Clec4a4*^−/−^ mice showed more prominent proliferation and secretion of IFN-γ as well as the generation of T_H_1 cells than those from WT mice (Fig. 6g–i). Similarly, *Clec4a4*^−/−^ mice had the enhanced Ag-specific proliferation and production of IFN-γ and IL-17 as well as the augmented generation of T_H_1 cells and T_H_17 cells compared with WT mice following immunization with CpG-B plus OVA (Supplementary Fig. 8d–f).

Taken together, these results indicate that the deficiency of Clec4A4 potentiates the responses of Ag-specific CD4^+^ T_eff_ cells *in vivo*.

### Clec4A4 reduces the responses of CD8^+^ T cells

**Figure 7:**
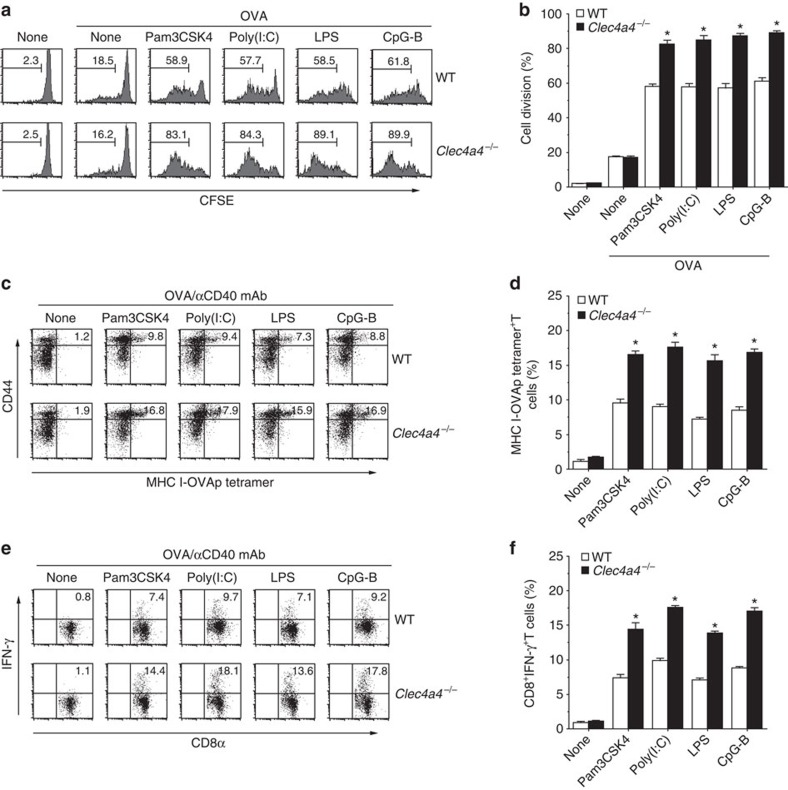
Deficiency of Clec4A4 promotes Ag-specific CD8^+^ T-cell responses *in vivo*. (**a**,**b**) CFSE-labelled CD45.1^+^OT-I CD8^+^ T cells were transferred into WT mice (*n*=6) and *Clec4a4*^−/−^ mice (*n*=6), and then the mice were immunized with OVA protein in combination with or without the indicated TLR ligands. Ag-specific division of CD45.1^+^OT-I CD8^+^ T cells was analysed 3 days after the immunization by flow cytometry. (**a**) Data are presented by a histogram, and numbers represent the proportion of CFSE dilution among gated CD45.1^+^OT-I CD8^+^ T cells in each quadrant. (**b**) Data are the mean percentage of positive cells±s.d. from six individual samples in a single experiment. (**c**–**f**) WT mice (*n*=6) and *Clec4a4*^−/−^ mice (*n*=6) were immunized with the indicated TLR ligands, anti-CD40 mAb, and OVA protein. At 6 days after the immunization, splenocytes were analysed for the generation of MHC I-OVA tetramer^+^CD44^high^CD8^+^ T cells (**c**,**d**) and for intracellular IFN-γ-producing CD8^+^ T cells (**e**,**f**) by flow cytometry. Data are presented by a dot plot (**c**,**e**) and numbers represent the proportion of MHC I-OVA tetramer^+^CD44^high^ cells (**c**) and IFN-γ^+^ cells (**e**) among gated CD8^+^ T cells in each quadrant. (**d**,**f**) Data are the mean percentage of positive cells±s.d. from six individual samples in a single experiment. *P<0.01 compared with WT mice (analysis of variance (ANOVA), Bonferroni's multiple comparison test). All data are representative of at least three independent experiments.

We addressed the influence of the deficiency of Clec4A4 on the capacity of CD8α^−^ cDCs to activate of CD8^+^ T cells through the cross-presentation of soluble Ag *in vivo*. On immunization with OVA protein plus various TLR ligands, *Clec4a4*^−/−^ mice displayed significant enhancement of Ag-specific proliferation of adoptively transferred OT-I CD8^+^ T cells compared with WT mice (Fig. 7a,b; Supplementary Fig. 9a).

To examine the influence of the deficiency of Clec4A4 on the induction of CTLs through the cross-presentation of soluble Ag, we quantified the generation of Ag-specific CD8^+^ T cells based on binding with the MHC I-OVA tetramer and intracellular expression of IFN-γ 6 days after immunization with OVA protein combined with various TLR ligands and anti-CD40 mAb (Fig. 7c–f; Supplementary Fig. 9b,c). *Clec4a4*^−/−^ mice had more marked augmentation in the induction of MHC I-OVA tetramer^+^CD44^high^CD8^+^ T cells and CD8^+^IFN-γ^+^ T cells than WT mice.

Collectively, these results indicate that the deficiency of Clec4A4 promotes the ability of CD8α^−^ cDCs to activate CD8^+^ T cells through cross-presentation *in vivo*.

### Clec4A4 ameliorates experimental autoimmune encephalitis

**Figure 8:**
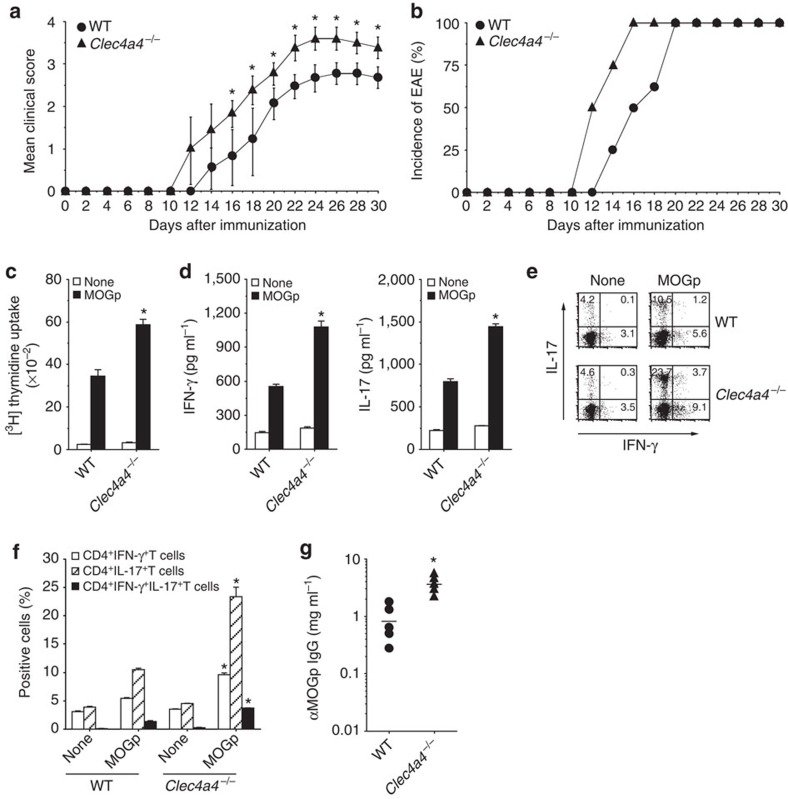
Deficiency of Clec4A4 aggravates EAE. WT mice (*n*=10) and *Clec4a4*^−/−^ mice (*n*=10) were immunized with CFA plus MOGp. (**a**,**b**) The disease severity of each mouse was scored, and mean clinical score±s.d. (**a**) and disease incidence (**b**) at the indicated times were plotted from 10 individual mouse in a single experiment. (**c**–**f**) At 14 days after the immunization, Spl CD4^+^ T cells were cultured with WT CD11c^+^ DCs in the presence or absence of MOGp for the measurement of proliferative responses by [^3^H]thymidine incorporation (**c**) and production of IFN-γ (**d**, left panel) and IL-17 (**d**, right panel) by ELISA. Data are the mean±s.d. from 10 individual samples in a single experiment. (**e**,**f**) Intracellular production of IFN-γ and IL-17 in the cultured CD4^+^ T cells was analysed by flow cytometry. (**e**) Data are presented by a dot plot, and numbers represent the proportion of IFN-γ^+^ cells and IL-17^+^ cells among gated CD4^+^ T cells in each quadrant. (**f**) Data are the mean percentage of positive cells±s.d. from 10 individual samples in a single experiment. (**g**) Serum MOGp-specific IgG production was measured at 22 days after immunization by ELISA. Data are the mean±s.d. from 10 individual samples in a single experiment. *P<0.01 compared with WT mice (analysis of variance (ANOVA), Bonferroni's multiple comparison test). All data are representative of at least three independent experiments.

We assessed the impact of the deficency of Clec4A4 on the initiation and progression of experimental autoimmune encephalitis (EAE). Immunization of WT mice with myelin oligodendrocyte glycoprotein_35–55_ peptide (MOGp) together with CFA elicited EAE (Fig. 8a,b), which was accompanied by the generation of Ag-specific CD4^+^ T-cell proliferation, and the induction of T_H_1 cells and T_H_17 cells following *ex vivo* recall antigenic stimulation, as well as the secretion of anti-MOGp-specific IgG (Fig. 8c–g). In contrast, *Clec4a4*^−/−^ mice showed more progressive development of EAE than WT mice (Fig. 8a,b). Furthermore, *Clec4a4*^−/−^mice displayed a higher Ag-specific proliferation of CD4^+^ T cells than WT mice (Fig. 8c). Indeed, CD4^+^ T cells from *Clec4a4*^−/−^ mice not only exhibited more potent production of IFN-γ and IL-17 but also had a higher frequency of T_H_1 cells and T_H_17 cells than WT mice (Fig. 8d–f). On the other hand, *Clec4a4*^−/−^ mice showed higher production of anti-MOGp-specific IgG than WT mice (Fig. 8g).

Collectively, the deficiency of Clec4A4 aggravates the development of T-cell-mediated autoimmune disease through the excessive activation of CD8α^−^ cDCs.

### Clec4A4 suppresses host protection against bacterial infection

To determine the role of Clec4A4 in the host protection against bacterial infections through the regulation of the function of CD8α^−^ cDCs, we examined the host protective immune responses against *Listeria monocytogenes* expressing OVA (*LM*-OVA)^25^. While WT mice succumbed to bacterial infection within 11 days, *Clec4a4*^−/−^ mice showed an enhanced survival rate (*P*<0.01), which was associated with significant reduction in splenic bacterial burden, and prominent elevation in serum productions of IL-6, IL-12p40 and IFN-γ (Supplementary Fig. 10a–c). Furthermore, *Clec4a4*^−/−^ mice displayed the enhanced anti-bacterial responses of CD4^+^ T cells following bacterial infection when compared with WT mice (Supplementary Fig. 10d–f).

Collectively, these results indicate that the deficiency of Clec4A4 promotes anti-bacterial protective immune responses through the augmentation of the function of CD8α^−^ cDCs unless the inflammation causes the microbial septic shock.

## Discussion

As described in this manuscript, our biochemical and genetic results clearly demonstrate that Clec4A4 acts as a unique ‘regulatory CLR' for the TLR-mediated activation of CD8α^−^ cDCs that is critical for control of the magnitude and quality of innate and adaptive immune responses. To our knowledge, our findings are the first describing that Clec4A4 known as a DC-specific surface marker molecule suppresses the cellular activation and maturation of CD8α^−^ cDCs, while most PRRs expressed on DCs reportedly act as activation receptors^1^,^2^,^3^. Furthermore, the Clec4A4-mediated regulation of CD8α^−^ cDCs was shown to impact on immune responses *in vivo*.

The family of Clec4As is the only group of classical CLRs with an ITIM in their cytoplasmic tail^17^,^26^. In sharp contrast to Clec4A4, Clec4A2 (DCIR1) is expressed on a various immune cells, such as B cells, monocytes/macrophages and DCs^17^,^22^,^27^. It has been shown that the triggering of human Clec4A with specific mAb not only elicits its internalization to endosomal/lysosomal compartments leading to Ag presentation, but also results in inhibition of the TLR9-mediated secretion of type I IFN on human pDCs and the TLR8-mediated production of proinflammatory cytokines on human cDCs, although which signalling pathway is elicited after the ligation of Clec4A, leading to the inhibition of TLR signalling, remains unresolved^17^,^28^,^29^. Here we show that retroviral transfection of Clec4A4 into cDCs impairs TLR-mediated cytokine production, possibly through suppression of the activation of MyD88- or TRIF-dependent activation of NF-κB, MAPKs and IRFs. Consistently, deficiency of Clec4A4 specifically leads to the hyperresponsiveness of CD8α^−^ cDCs to various TLR ligands for the secretion of cytokines mediated by the augmented activation of NF-κB, MAPKs and IRFs. Thus, our findings strongly suggest that Clec4A4 generates an inhibitory signal for the TLR-mediated downstream cascades to abrogate the activation of CD8α^−^ cDCs.

Previous studies have shown that the phosphorylated ITIM in human Clec4A is able to interact with SHP-1 and SHP-2 using pervanadate treatment^30^ or a peptide containing the phosphorylated form of ITIM^31^, although their physiological relevance remains unclear. We demonstrated that Clec4A4 mutant lacking ITIM in cytoplasmic tails had no inhibition on the TLR-mediated cytokine production in cDCs, suggesting that the inhibitory effect of Clec4A4 on the TLR-mediated activation of cDCs directly involves the ITIM-mediated intracellular signalling. Furthermore, Clec4A4 constitutively associated with SHPs under steady-state phosphorylation of its ITIM, and the ligation of Clec4A4 not only potentiated its phosphorylation status, but also enhanced the recruitment of these phosphatases and their functions. As the deficiency of SHPs reportedly amplified the TLR-mediated downstream signalling cascades for the enhanced production of IFN-I and proinflammatory cytokines, SHP-1 has been shown to block the MyD88-dependent cytokine secretion by suppressing the activation of NF-κB, MAPK, IRAK1, IKK-α and IKK-β mediated through directly binding and dephosphorylating them, whereas SHP-2 has been shown to inhibit the TLR3/4-mediared cytokine production in TRIF-dependent signalling due to the direct interaction with TBK1 (refs 4, 32, 33, 34). Therefore, the molecular inhibitory machinery in the Clec4A4-mediated suppression of TLR ligand-induced cytokine production could involve the association of SHP-1 or SHP-2 with the phosphorylated ITIM in Clec4A4, leading to the impairment of MyD88- or TRIF-dependent activation of the downstream signalling pathway.

Analysis of Clec4A4 mutant lacking extracellular portion suggests that the excessive expression of membrane-anchored ITIM might be insufficient for the suppressive effect on the TLR-mediated activation of cDCs when extracellular portion of Clec4A4 is lacking. Furthermore, a glycosylation mutant of Clec4A4 lacking the *N*-glycosylation site in the CRD, which possibly prevented the formation of self-interaction, reduced the inhibitory effect. Thus, the self-interaction of Clec4A4 in *cis* and/or *trans* through the binding of its CRD with oligosaccharide resides on glycans could be required for the suppressive effect on the TLR-mediated activation of cDCs.

Similar to the proposed concept for the masking of Siglecs^35^, the potential interaction of Clec4A4 with glycans present on Clec4A4 itself and *cis* ligands existed on neighbouring glycoproteins could occupy its CRD because we clearly demonstrated that the steady-state phosphorylation of ITIM in Clec4A4 in cDCs. Indeed, we showed that the soluble form of Clec4A4 specifically bound to Man, Fuc, GlcNAc and GalNAc moieties on glycans and Clec4A4-expressing cDC transfectants, while it also bound to their control transfectants to a lesser extent. Thus, it is intriguing to hypothesize that Clec4A4 constitutively associates with itself in addition to other adjacent glycoproteins (for example, SIGNR1) mediated through the binding of CRD with oligosaccharide resides on glycans, and the inhibitory signalling via ITIM in Clec4A4 could potentially occur under steady-state conditions, resulting in lowering of the responsiveness of CD8α^−^ cDCs to TLR-mediated activation.

Different from our observation on the suppressive role of Clec4A4 in the TLR-mediated activation of CD8α^−^ cDCs, the deficiency of Clec4A2 reportedly did not affect the response of BMDCs to LPS stimulation^36^, despite the fact that these two Clec4As share similar extracellular domain and cytoplasmic portions. It remains unclear how distinct Clec4A4s lead to different cellular responses, the kinetics, affinity and specificity of glycan binding, or the valency of engagement of each Clec4A, as well as how cell-type-specific expression potentially accounts for the distinct signalling through the ITIM-mediated regulation of cell function.

Whereas various immune cells, including DCs and non-haematopoietic cells, have been reported to express various TLRs to respond to each ligand^37^, the contribution of CD8α^−^ cDCs to the TLR-mediated responses and their regulatory mechanism *in vivo* remains unclear. In line with the augmented TLR-mediated cytokine production by *Clec4a4*^−/−^ CD8α^−^ cDCs, analysis of *in vivo* responsive to TLR ligands and bacterial peritonitis revealed that *Clec4a4*^−/−^ mice exhibited hyperinflammatory response associated with the excessive production of cytokines. These findings suggest that Clec4A4 increases the threshold of responsiveness of CD8α^−^ cDCs to TLR ligands as well as other pathogen-associated molecular patterns to prevent excessive detrimental to inflammation.

Although there have been several reports describing the role for CD8α^−^ cDCs in the induction of the responses of CD4^+^ T cells *in vivo* by the Ag targeting to this DC subset via 33D1 mAb^7^,^20^, how CD8α^−^ cDCs instruct and regulate the responses of CD4^+^ T cells *in vivo* remains unclear. Our *in vitro* analysis showed that the deficiency of Clec4A4 promoted the ability of CD8α^−^ cDCs to generate Ag-specific T_H_1/T_H_17 cells. Furthermore, the deficiency of Clec4A4 not only enhanced Ag-specific priming of CD4^+^ T cells but also augmented CD4^+^ T_eff_-cell responses *in vivo*. These observations strongly suggest that the deficiency of Clec4A4 leads to enhancement of the capacity of CD8α^−^ cDCs to upregulate the expression of MHC and costimulatory molecules, and to produce cytokines after the ligation of TLRs, resulting in the promoted development of CD4^+^ T_eff_ cells from naive CD4^+^ T cells *in vivo* under inflammatory conditions. Thus, Clec4A4 could regulate APC function of CD8α^−^ cDCs for tight control of the direction of the responses of CD4^+^ T_eff_ cells *in vivo*.

CD8α^+^ cDCs have reportedly shown to be superior to CD8α^−^ cDCs to prime CD8^+^ T cells for the generation of CTL response through cross-presentation, possibly owing to the differences in expression of proteins associated with Ag processing for MHC I presentation^1^,^7^,^8^. Notably, the deficiency of Clec4A4 caused a further increase in the priming of Ag-specific CD8^+^ T cells and induction of CTLs *in vivo* when soluble Ag was immunized, demonstrating the potential cross-presentation capacity of CD8α^−^ cDCs for the efficient generation of CTLs. Therefore, Clec4A4 could strictly suppress the TLR-mediated amplification of the expression of several proteins involved in cross-presentation to activate CD8^+^ T cells in CD8α^−^ cDCs under pathophysiological conditions.

It has been shown that *Clec4a2* (*Dcir1)*^−/−^ mice exhibited an exacerbation of the pathogenesis in collagen-induced arthritis (CIA), possibly due to the excessive expansion of CD11c^+^ DCs^36^. Furthermore, the expression of CD80, CD86 and MHC II on CD11c^+^ DCs in CIA-induced *Clec4a2*^−/−^ mice was at a normal level for their steady-state conditions, indicating that DCIR1 could not be involved in the maturation and activation of CD11c^+^ DCs^36^. On the other hand, our results demonstrated that *Clec4a4*^−/−^mice displayed progression of the pathogenesis of EAE, which is accompanied by the enhanced generation of MOG-specific T_H_1 cells and T_H_17 cells as well as massive production of anti-MOGp-specific IgG. Therefore, in sharp contrast to the suppressive role of Clec4A2 in the expansion of CD11c^+^ DCs for the development of autoimmune diseases, Clec4A4 could specifically downregulate the maturation and activation of CD8α^−^ cDCs rather than their expansion, leading to impairment of the pathogenic responses of CD4^+^ T_eff_ cells, which is responsible for amelioration of the pathogenesis of autoimmune diseases.

While cDCs have reportedly been involved in host defenses against bacterial infections mediated through the recognition of pathogen-associated molecular patterns by several activating PRRs^5^,^38^, the role of CLRs in the abortive activation of cDCs leading to the inhibition of anti-microbial immunity remains to be understood. On bacterial infection, the deficiency of Clec4A4 not only led to the augmentation of anti-bacterial CD4^+^ T_eff_ cell-responses, but also displayed the prominent elimination of the invaded bacteria, resulting in the reduction of the mortality. These observations led us to hypothesize that the interaction of the CRD of Clec4A4 with the glycan present on microbes also cause the impaired activation of CD8α^−^ cDCs, which hampers anti-microbial host defense.

In conclusion, we described that Clec4A4 constitutes a CLR endowed with critical negative regulatory function in CD8α^−^ cDCs, in which it prevents the induction of exacerbated inflammation and pathological T-cell responses, and therefore maintains immune homeostasis and prevents immunopathogenesis. Conversely, Clec4A4 also contributes to the subversion of anti-microbial host protective immune responses mediated through the abortive activation of CD8α^−^ cDCs, which is linked to the microbial evasion from immune surveillance. Thus, our findings proposed the concept that ‘regulatory CLR' binding to endogenous ligands on cDCs and pathogens plays an crucial role in immune homeostasis and host defense. Targeting this unique CLR with antibody (Ab) may constitute new therapies for autoimmune and inflammatory disorders as well as infectious diseases and cancer.

## Methods

### Mice

The following 8- to 12-week-old female mice were used in this study: C57BL/6 mice (Japan Clea), B6.CD45.1^+^OT-I TCR transgenic mice harbouring OVA-specific CD8^+^ T cells^6^,^24^, and B6.CD 45.1^+^OT-II TCR transgenic mice harbouring OVA-specific CD4^+^ T cells^6^,^24^, and B6.*Clec4a4*^−/−^ mice as described below. All mice were bred and maintained in specific pathogen-free conditions in the animal facility at University of Miyazaki in accordance with institutional guidelines of the Animal Experiment Review Board.

### Generation of *Clec4a4* ^−/−^ mice

The targeting vector for *Clec4a4*^−/−^mice was constructed in the pBluescript vector by using a 4.0-kb genomic fragment (left arm) upstream of *Clec4a4* exon 1 and a 2.0-kb genomic fragment (right arm) downstream of exon 2 cloned from a modified bacterial artificial chromosome clone, RP23-265M17 (Children's Hospital Oakland Research Institute), containing the complete *Clec4a4* gene (gene symbol *Clec4a4*). The left arm was generated by PCR using the following oligonucleotides: left arm forward (5′-CGC*CTCGAG*GTGATGAATCAAAGATTTAACAGAATGTA-3′) and left arm reverse (5′-CGC*GTCGAC*TTCAAGAAAAGACCTGCCTCCCTCAGAAAGCACAA-3′). The 4.0-kb PCR fragment was digested with XhoI and SalI, and ligated into each site of pBluescript. The right arm was generated by PCR using the following oligonucleotides: right arm forward (5′-CGC*GTCGAC*GTAAGTATCCTGCACACATCAATGGGCCTTGTCTG-3′) and right arm reverse (5′-CGC*ACTAGT*GTTCCTCAGAAAATTGGACATATTACTAC-3′), digested with SalI and SpeI, and ligated into each site of the targeting vector. Each of the 5′ and 3′ primers was also tagged (indicated in italics) with XhoI and SalI sites for the left arm or SalI and SpeI sites for the right arm, respectively. A SalI restriction site was engineered in place of the start codon in exon 1. The *pIRES2-EGFP-loxP-Cre/Neo*^*r*^*-loxP* auto-deleter cassette^39^ was cloned into the SalI site inserted into the targeting vector. Finally, the targeting construct was abutted to a PMC1-DTa negative-selection cassette and linearized. The linearized targeting construct was introduced by electroporation into C57BL/6-derived Bruce4 recombinant embryonic stem cell and neomycin-resistant clones were first screened for homologous recombination by PCR utilizing a pair of the following oligonucleotides corresponding to a sequence outside of the 5′ left arm and to the EGFP site: Primer 1: 5′-GAGTACCTTCTAGGTCTATGTGACTTGACT-3′, and Primer 2: 5′-ATATAGACGTTGTGGCTGTTGTAGTTGTA-3′. EcoRV-digested genomic DNA of positive clones was then screened by Southern blotting with a 3′ external single-copy probe corresponding to a 0.507-kb fragment (Supplementary Fig. 3f), which was amplified by PCR using the oligonucleotides 5′-TTGGTGAAAATTAAAATCACATTCA-3′ and 5′- TGGCATTATAATTAGCTGACACTGA-3′. When tested on EcoRV-digested DNA, it hybridized either to a 8.3-kb WT fragment or to a 7.6-kb recombinant fragment. Embryonic stem cell clones bearing the correctly targeted locus were injected into BALB/c blastocysts, and chimeric male offspring, in which the autodeleter cassette was self-excised during the male germline transmission, were mated with female C57BL/6 mice to obtain heterozygotes, which were then crossed to obtain homozygotes. Transmission of the targeted allele was confirmed by PCR with Primer 1 and Primer 2 as described above. CRE-mediated deletion of the floxed Neo^r^ cassette can be visualized by the presence of a 6.3-kb fragment using EcoRV-digested DNA hybridized with the 3′ external single-copy probe as described above. The mutant mice were cross-mated for more than nine generations with C57BL/6 mice, and 8- to 12-week-old female *Clec4a4*^+/+^ littermates were used as WT mice.

### Cell isolation

To prepare single-cell suspensions from spleen, PLNs and MLNs, tissue samples were digested with collagenase type III (Worthington Biochemical) at 37 °C for 20 min, and were ground between glass slides. Splenocytes were treated with red blood cell lysis buffer (Sigma-Aldrich) before suspension. BM cells were flushed from the femurs and tibias. Single-cell suspensions were obtained by forcing through a 100-μm cell strainer (BD Biosciences). CD4^+^ T cells and CD8^+^ T cells were purified from the spleen with mouse CD4 T-lymphocyte Enrichment Set-DM and mouse CD8 T-lymphocyte Enrichment Set-DM (both from BD Biosciences). CD11c^+^ DCs were purified by AutoMACS with mouse CD11c (N418) Microbeads (Miltenyi Biotec). For the preparation of CD11c^high^CD8α^−^ cDCs, splenocytes were depleted of T cells, B cells and granulocytes with biotin-conjugated Ab cocktail and anti-biotin Microbeads from CD4^+^ Dendritic Cell Isolation kit (Miltenyi Biotec) according to the manufacturer's directions with some modifications, and then subjected to purification using mouse CD11c (N418) Microbeads. In some experiments, CD11c^+^ DCs were sorted into CD11c^high^CD8α^−^ cDCs with high purity (each >99%) using a FACSAriaII cell sorter (BD Biosciences) with fluorescein-conjugated mAbs. BMDCs were generated by culturing BM cells with granulocyte–macrophage colony-stimulating factor (GM-CSF, 20 ng ml^−1^, Wako Pure Chemical Industries) for 8 days^40^,^41^.

### Retrovirus-mediated gene transfer

Total RNA from CD8α^−^ cDCs (10^6^) was extracted with TRIzol (Life Technologies), and cDNA was synthesized with oligo(dT)_20_ as a primer using the SuperScript III First-Strand Synthesis System for RT–PCR kit (Life Technologies). The full-length cDNA of C terminus FLAG-tagged *Clec4a4* (NCBI: AY397673) was amplified by PCR using GoTaq Green Master Mix (Promega) with a pair of specific primers (5′-CGC*GGATCC*GCCGCCATGGCTTCAGAAATC-3′ and 5′-CGC*CTCGAG*TCA*CTTGTCATCGTCGTCCTTGTAGTC*TAAGTATATTTTCTT-3′). Each of the 5′ and 3′ primers was also tagged (indicated in italics) with BamH1 and XhoI sites, while kosac sequence or FLAG sequence was indicated with an underline or an underline in italics. The PCR product was subcloned into pCR4-TOPO using TA TOPO Cloning Kit for Sequencing (Life Technologies) and the nucleotide sequence was confirmed with an ABI3100xl automated sequencer (Applied Biosystems) and the fluoresceinated dye terminator cycle sequencing method. Alternatively, the DNA sequence of Clec4A4_ΔI5–V10_, Clec4A4_ΔY68–L236_ or Clec4A4_N186Q_, which were tagged with BamH1 and XhoI sites in 5′ and 3′ terminals, was custom-made using GeneArt (Life Technologies). After restriction enzyme digestion, the DNA sequence was cloned into the sites of the pMX-IRES-GFP vector^41^,^42^, and was transfected into a retroviral packaging cell line, Phoenix^41^,^42^, with LipofectAMINE Plus Reagent (Life Technologies). The culture supernatant of Phoenix after 24 h of culture was collected and centrifuged at 8,000*g* for 16 h at 4 °C to concentrate the virus. The retroviral vector was transfected into BM for the generation of BM-DCs as described above or DC2.4 cell lines together with DOTAP Liposomal Transfection Reagent (Roche) by centrifugation at 2,000*g* for 1 h at 32 °C, and transfectants were subsequently collected to examine GFP expression by using FACSAriaII cell sorter.

### Generation of human IgG Fc fusion protein

The DNA sequences containing the extracellular domain lacking the signal sequence and intracellular domain of *Clec4a4* (NCBI: AAR31149 amino acids: 68–236) were custom-made using GeneArt, and the 5′ and 3′ ends of the synthesized DNA sequences were also tagged with EcoRI and BglII sites, respectively. After restriction enzyme digestion, the synthesized DNA sequence was cloned into the sites of pFUSE-hIgG2-Fc2 (Invivogen), and transfected into FreeStyle 293-F cells (Life Technologies) using 293fectin Transfection Reagent (Life Technologies) according to the manufacturers' instructions. huIgFc or Clec4A4-huIgFc fusion protein was purified from the culture supernatant using HiTrap Protein G HP (GE Healthcare Life Sciences), and the purified fusion protein was separated by 10% SDS–PAGE under non-reducing or reducing conditions, respectively, and stained with a Silver Stain kit (Bio-Rad Laboratories) to confirm the protein products.

### Flow cytometry

Cells were stained with fluorescein-conjugated mAbs (1:100) to mouse CD3ɛ (cat#553066, 145-2C11), CD4 (cat#553046, RM4-5), CD8α (cat#553032, 53-6.7), CD11c (cat#550261, HL3), CD40 (cat#553723, 3/23), CD44 (cat#559250, IM7), CD45.1 (cat#558701, A20), CD49b (cat#553857, DX5), CD80 (cat#553768, 16-10A1), CD86 (cat#553691, GL1), I-A/I-E (cat#557000, M5/114.15.2), B220 (cat#553089, RA3-6B2), H-2K^b^ (cat#553569, AF6-88.5), Vα2 TCR (cat#553289, B20.1), IFN-γ (cat#554411, XMG1.2), IL-17A (cat#559502, TC11-18H10), isotype-matched control mAb (cat#550003/559940, 187.1; BD Biosciences), dendritic cell marker (cat#12-5884, 33D1; eBioscience), CD11b (cat#101206, M1/70; Biolegend), Siglec-H (cat#130-102-229, JF05-1C2.4.1; Miltenyi Biotec), and H-2K^b^ OVA tetramer (cat#TS-5001-1C; MBL). For the intracellular expression of cytokines, cells were incubated for 4 h with phorbol 12-myristate 13-acetate (50 ng ml^−1^; Sigma-Aldrich) plus ionomycin (500 ng ml^−1^; Sigma-Aldrich) or OVA_257–264_ peptide (SIINFEKL; 10 μM) plus GolgiPlug (BD Biosciences) during the final 2 h. Subsequently, the cells were resuspended in Fixation-Permeabilization solution (BD Cytofix/Cytoperm kit; BD Biosciences) and intracellular cytokine staining was carried out according to the manufacturer's directions. For staining with recombinant fusion proteins, cells were stained with huIgFc (1 μg), Clec4A4-huIgFc (1 μg), SIGNR1-huIgFc (cat#1836-SR, 1 μg), or Clec9A-huIgFc (cat#6049-CL, 1 μg; both from R&D Systems) followed by anti-human IgG-PE (cat#12-4998-82; eBiosciences). Fluorescence staining was analysed with a FACSCalibur flow cytometer and CELLQuest Software (both from BD Biosciences).

### Culture of CD11c^+^ DCs

CD11c^high^CD8α^−^ cDCs were cultured with or without Pam3CSK4 (1 μg ml^−1^), poly(I:C) (50 μg ml^−1^), LPS (1 μg ml^−1^), or CpG-B (0.1 μM) for 18 h in 48-well culture plates (BD Bioscience). Similarly, BMDCs expressing mock-GFP, Clec4A4-GFP, Clec4A4_N186Q_-GFP, Clec4A4_ΔY68–L236_-GFP, or Clec4A4_ΔI5–V10_-GFP were cultured with or without LPS (1 μg ml^−1^) or CpG-B (0.1 μM) for 18 h in 48-well culture plates. Alternatively, DC2.4, DC2.4-expressing mock-GFP or DC2.4-expressing Clec4A4-GFP (5 × 10^5^) were cultured with or without Pam3CSK4 (1 μg ml^−1^), poly(I:C) (50 μg ml^−1^), LPS (0.1 μg ml^−1^), or CpG-B (0.1 μM) for 18 h in 48-well culture plates. The culture supernatants were collected and stored at −80 °C until assayed for cytokines.

### Detection of cytokines

Culture supernatants and sera were assayed for IFN-β (PBL), TNF-α, IL-6, IL-12p40 (eBioscience), IL-17A and IFN-γ (Biolegend) using enzyme-linked immunosorbent assay (ELISA) kits according to the manufacturers' instructions.

### Immunoblotting

DC2.4, DC2.4-expressing mock-GFP and DC2.4-expressing Clec4A4-GFP (2 × 10^6^) were stimulated or not stimulated with LPS (0.1 μg ml^−1^) for the period indicated. Alternatively, BMDCs expressing mock-GFP or Clec4A4-GFP as well as CD8α^−^ cDCs (2 × 10^6^) were stimulated or not stimulated with LPS (1 μg ml^−1^) or CpG-B (1 μg ml^−1^) for the period indicated. Total lysate was analysed by SDS–PAGE, and blots were probed with Ab (1:1,000) for NF-κB p65 (cat#8242, D4E12), ERK (cat#9102), JNK (cat#9252), p38 (cat#9212), IRF-3 (cat#4302, D83B9) (Cell Signaling Technology) and IRF-7 (cat#sc-9083, H-246; Santa Cruz Biotechnology) or the phosphospecific Ab (1:1,000) for NF-κB p65 (cat#3033, 93H1), ERK (cat#9101), JNK (cat#9251), p38 (cat#9211), IRF-3 (cat#4947, 4D4G) and IRF-7 (cat#14767; Cell Signaling Technology) followed by horseradish peroxidase (HRP)-conjugated goat anti-rabbit IgG (cat#7074, 1:1,000; Cell Signaling Technology). In some experiments, BMDCs expressing mock-GFP or Clec4A4-GFP as well as DC2.4, DC2.4-expressing mock-GFP and DC2.4-expressing Clec4A4-GFP (2 × 10^6^) were pretreated with or without the cross-linked anti-FLAG M2 mAb (cat#F1804, 10 μg ml^−1^; Sigma-Aldrich) with secondary goat anti-rabbit IgG (cat#111-005-003, 100 μg ml^−1^; Jackson ImmunoResearch) for 30 min. Then, cells were stimulated or not stimulated with LPS (1 μg ml^−1^) or CpG-B (1 μg ml^−1^) for 30 min. Subsequently, the immunoprecipitate with anti-FLAG M2 mAb (F1804, 1:100), anti-SHP-1 mAb (cat#sc-30810, G-20, 1:100) or anti-SHP-2 mAb (cat#sc-280, C-18, 1:100) (Santa Cruz Biotechnology) obtained from total cell lysate was analysed by SDS–PAGE, and blots were probed with anti-phosphotyrosine (p-Tyr) mAb (cat#sc-7020, PY99, 1:1,000; Santa Cruz Biotechnology) followed by HRP-conjugated goat anti-mouse IgG (cat#1031-05, 1:1,000; SouthernBiotech), anti-FLAG M5 mAb (cat#1031-05, 1:1,000; Sigma-Aldrich), anti-SHP-1 mAb (cat#3759, C14H6, 1:1,000) or anti-SHP-2 mAb (cat#3397, D50F2, 1:1,000) (Cell Signaling Technology) followed by HRP-conjugated goat anti-rabbit IgG (cat#7074). The blot was visualized by ChemiDoc XRS+ Image System (BIO-RAD) with an ECL Plus Western Blotting Detection System (GE Healthcare). Images have been cropped for presentation. Full-size images are presented in Supplementary Figs 11, 12,13 and 14.

### SHP assay

Analysis of protein tyrosine phosphatase activity was described as previously^43^. Briefly, the immunoprecipitate with anti-FLAG M2 mAb (prepared in lysis buffer without sodium orthovanadate) were incubated at 37 °C for 30 min in 200 μl of phosphatase buffer (62 mM HEPES, pH 5.0), 6.25 mM EDTA and 12.5 mM DTT) containing 25 mM *p*-nitrophenylphosphate (Sigma-Aldrich). Reactions were terminated by addition of 0.8 ml of 1 N NaOH, and the absorbance measured at 410 nm by spectrophotometry.

### Immunohistochemical analysis

Spleen was embedded in OCT compound (Sakura Finetechnical) and frozen in liquid N_2_. The tissue segments were sectioned with a cryostat at 8 μm. Frozen sections were fixed in cold acetone and blocked in TNB buffer (PerkinElmer Life Science) containing 5% normal rat serum. To block endogenous biotin, the sections were further treated with the Streptavidin/Biotin Blocking Kit (Vector Laboratories), and endogenous peroxidase activity was quenched with 1% H_2_O_2_. The primary Abs were anti-CD19-FITC mAb (cat#553785, 1D3; BD Biosciences), anti-mouse dendritic cell marker-biotin mAb (cat#130-101-843, 33D1; Miltenyi Biotec), anti-CD11c mAb-biotin mAb (cat#553800, HL3; BD Biosciences) and anti-CD3ɛ-APC mAb (cat#100312, 145-2C11; Biolegend). The CD19 signal was amplified by incubation with Alexa Fluor 488-conjugated anti-rat IgG (cat#A11006; Life Technologies). The 33D1 and CD11c staining was revealed with a TSA signal amplification kit (PerkinElmer Life Science) according to the manufacturer's instructions. The sections were incubated with Streptavidin-HRP (PerkinElmer Life Science) followed by Tyramide-Cy3 conjugate. At the end of the staining, slides were washed and mounted with Vectashield (Vector Laboratories). The stained slides were examined with a BIOREVO fluorescence microscope (BZ-9000; KEYENCE).

### Glycan binding assay

The preparation of NGLs constructed with oligosaccharides (Man3, LNFP3, LNT and GlcNAc) and DPPG, asialo-GM2, and lactocerebrosides were described as previously^23^. For glycan binding assay, NGLs, asialo-GM2, and lactocerebrosides dissolved in isopropanol (100 pM) were coated to the wells of the ELISA plate, and the wells were dried at 37 °C, and washed with PBS. After blocking with 1% BSA (Fraction V, Sigma-Aldrich) in PBS for 1 h at room temperature, huIgFc, Clec4A4-huIgFc, SIGNR1-huIgFc or Clec9A-huIgFc (5 μg ml^−1^) was added to the wells of the ELISA plate for at least 2 h at room temperature. After extensive washing with PBS, HRP-conjugated goat anti-human IgG (Fc specific; cat#A0170, 1:20,000; Sigma-Aldrich) was added to the wells. The reaction was developed with 3,3′,5,5′-tetramethylbenzidine as a substrate (Sigma Aldrich), and the absorbance was measured at 450 nm by microplate reader (iMark, BIO-RAD).

### Ag presentation assay

CD45.1^+^OT-I CD8^+^ T cells (10^5^) or CD45.1^+^OT-II CD4^+^ T cells (10^5^) were cultured with the irradiated (15 Gy) CD8α^−^ cDCs (1.25 × 10^3^–10^4^) in the presence of OVA_257–264_ peptide (1 pM–1 nM) or OVA_323–339_ peptide (ISQAVHAAHAEINEAGR; 1 nM–1 μM) for 3 days in 96-well flat-bottomed plates. Alternatively, CD4^+^ T cells (2 × 10^5^) were cultured with irradiated (15 Gy) CD11c^+^ DCs (2 × 10^4^) in the presence or absence of OVA protein (1 mg ml^−1^, Sigma-Aldrich) or MOG_35–55_ peptide (MEVGWYRSPFSRVVHLYRNGK; 1 μM) for 3 days in 96-well flat-bottomed plates (BD Biosciences). [^3^H]thymidine (GE Healthcare) incorporation was measured on day 3 for the last 18 h. In another experiment, the cells and the culture supernatants were collected to detect the production of cytokines.

### *In vivo* TLR stimulation

Mice were intraperitoneally (i.p.) injected with Pam3CSK4 (20 μg per mouse), poly(I:C) (50 μg per mouse), LPS (10 μg per mouse) or CpG-B (10 μg per mouse), and sera were collected at the indicated times. In some experiments, spleen was obtained from the mice 24 h after injection.

### Model for bacterial peritonitis

For bacterial peritonitis^38^, mice were injected i.p. with heat-killed (95 °C for 30 min) *E. coli* (DH5α, Life Technologies; 5 × 10^7^ per mouse). Survival was monitored at the indicated times for 60 h after injection and sera were collected 24 h after injection.

### *In vitro* CD4^+^ T-cell differentiation assay

For the differentiation of T_H_1 cells^44^, CD45.1^+^OT-II CD4^+^ T cells (2 × 10^5^) were cultured with CD8α^−^ cDCs (2 × 10^4^) in the presence or absence of Pam3CSK4 (1.25–1 μg ml^−1^), poly(I:C) (12.5–50 μg ml^−1^), LPS (0.25–1 μg ml^−1^) or CpG-B (0.025–0.1 μM) in combination with OVA_323–339_ peptide (1 μM), anti-IL-4 mAb (10 μg ml^−1^; 11B11, BD Biosciences) and recombinant mouse IL-2 (0.2 ng ml^−1^; Wako Pure Chemicals) for 3 days in 96-well flat-bottomed plates. For the differentiation of T_H_17 cells^44^, CD45.1^+^OT-II CD4^+^ T cells (2 × 10^5^) were cultured with CD8α^−^ cDCs (2 × 10^4^) in the presence or absence of TLR ligands as described above in combination with OVA_323–339_ peptide (1 μM), anti-IFN-γ mAb (10 μg ml^−1^; R4-6A2, BD Biosciences), anti-IL-4 mAb (10 μg ml^−1^; 11B11), recombinant mouse IL-2 (0.2 ng ml^−1^) and recombinant human transforming growth factor-β1 (10 ng ml^−1^; Wako Pure Chemicals) for 3 days in 96-well flat-bottomed plates. Analysis of the expression of IFN-γ or IL-17 among gated CD4^+^ T cells was performed by flow cytometry as described above.

### Adoptive transfer

CD45.1^+^OT-I CD8^+^ T cells or CD45.1^+^OT-II CD4^+^ T cells were labelled with CFSE (Molecular Probes; 2.5 μM) at 37 °C for 10 min, and washed twice with cold PBS. Subsequently, CFSE-labelled CD45.1^+^OT-I CD8^+^ T cells or CD45.1^+^OT-II CD4^+^ T cells (5 × 10^6^ per mouse) were intravenously injected into mice 24 h before the i.p. injection with or without OVA protein (50 μg per mouse) in combination with or without Pam3CSK4 (20 μg per mouse), poly(I:C) (50 μg per mouse), LPS (10 μg per mouse) or CpG-B (10 μg per mouse). After 1–3 days, the gated CD45.1^+^OT-I CD8^+^ T cells and CD45.1^+^OT-II CD4^+^ T cells in the spleen were analysed for CFSE dilution to detect the dividing cells by flow cytometry.

### Immunization

For the analysis of Ag-specific CD4^+^ T-cell responses, mice were immunized subcutaneously (s.c.) with OVA protein (100 μg per mouse) emulsified in CFA (Difco) or OVA protein (100 μg per mouse) plus CpG-B (50 μg per mouse) and the spleen was obtained 14 days after the immunization. For the generation of antigen-specific CTLs^6^,^24^,^45^, mice received an i.p. injection of Pam3CSK4 (20 μg per mouse), poly(I:C) (50 μg per mouse), LPS (10 μg per mouse) or CpG-B (10 μg per mouse) in combination with an i.p. injection of OVA protein (500 μg per mouse) plus of anti-CD40 mAb (10 μg per mouse; 1C10; eBioscience) and the spleen was obtained from the mice 3–9 days later.

### EAE induction

Mice were immunized s.c. with MOG_35–55_ peptide (200 μg per mouse, BEX) emulsified in CFA (500 μg of H37Ra) on day 0 and pertussis toxin (500 ng, CALBIOCHEM) i.p. on day 0 and again on day 2. Clinical scores were as follows: tail weakness, grade 1; hind limb weakness, grade 2; partial hind limb paralysis and weight loss, grade 3; paraplegia and incontinence, grade 4; quadriplegia and wasting, grade 5; and moribund, grade 6. In another experiment, mice were killed on day 14 after immunization to obtain the spleen. In some experiments, sera were obtained on day 22 after immunization, and assayed for MOG_35–55_ peptide-specific IgG^46^ using ELISA kits (ANASpec) according to the manufacturer's instructions.

### Bacteria and infection

*LM*-OVA were grown in brain–heart infusion broth (Merck). For bacterial infections^6^,^24^, mice were i.p. infected with *LM*-OVA (5 × 10^5^ c.f.u. per mouse). Survival was then monitored for 15 days or serum samples were collected 6 days after infection. For the determination of bacterial burden, splenic growth of *LM*-OVA was quantified by plating serial dilutions of homogenates on Oxford-Listeria-Selective-Agar (Merck) 6 days after infection, and the colonies were counted after an overnight incubation at 37 °C. In some experiments, the spleen was obtained from mice 6 days after infection.

### Statistical analysis

Data are expressed as the mean±s.d. The statistical significance of the differences between the values obtained was evaluated by one-way analysis of variance with Bonferroni's multiple comparison post-test and the Kaplan–Meier log-rank test. A *P* value of <0.01 was considered significant.

## Additional information

**How to cite this article:** Uto, T. *et al*. Clec4A4 is a regulatory receptor for dendritic cells that impairs inflammation and T-cell immunity. *Nat. Commun.* 7:11273 doi: 10.1038/ncomms11273 (2016).

## Supplementary Material

Supplementary Information Supplementary Figures 1-14
