# Supplementary material for: Clec4A4 is a regulatory receptor for dendritic cells that impairs inflammation and T-cell immunity
Source: Nat Commun. 2016 Apr 12;7:11273. doi: 10.1038/ncomms11273 (PMC4832068; doi:10.1038/ncomms11273)
Supplement: Supplementary Information — Supplementary Figures 1-14 [file ncomms11273-s1.pdf]

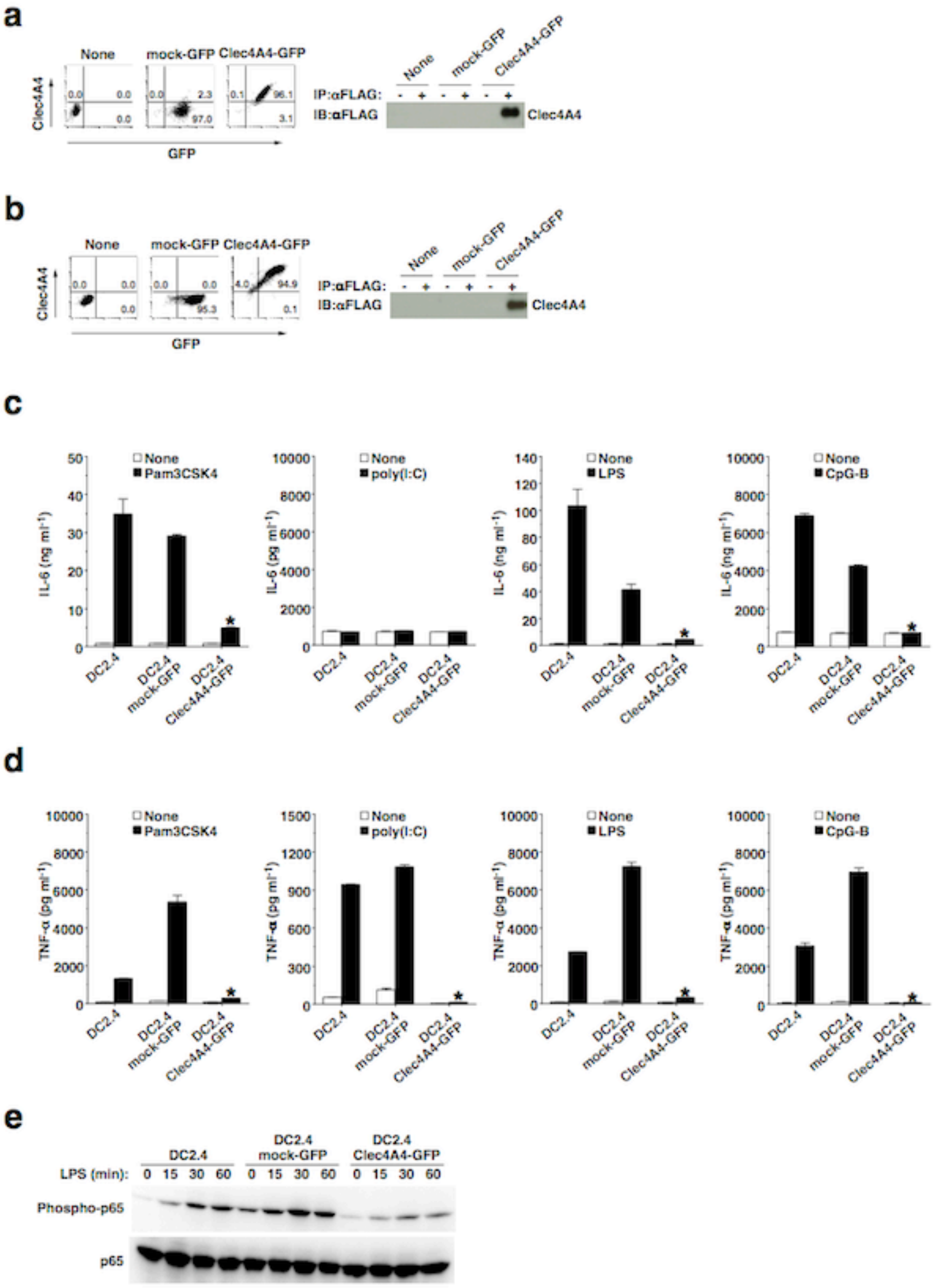

Supplementary Figure 1 Retroviral transduction of Clec4A4 suppresses the TLR-mediated activation of cDC cell line. (**a**,**b**) BMDCs (**a**) or DC2.4 (**b**) were transfected with or without pMX-IRES-GFP vector or pMX-Clec4A4-IRES-GFP vector. (Left panel) The expression of Clec4A4 and GFP was analyzed by flow cytometry. Data

are presented by a dot plot, and numbers represent the proportion in each quadrant. (Right panel) Total lysate were obtained from BMDCs or DC2.4 (None), BMDCs or DC2.4 expressing mock-GFP, and BMDCs or DC2.4 expressing Clec4A4-GFP, and the immunoprecipitate with anti-FLAG M2 mAb was analyzed using anti-FLAG M5 mAb. (**c,d**) DC2.4, DC2.4 expressing mock-GFP, and DC2.4 expressing Clec4A4-GFP were stimulated or not stimulated with the indicated TLR ligands, and the production of IL-6 (**c**) and TNF-α (**d**) was measured by ELISA. Data are the mean ± s.d. from three individual samples in a single experiment. *P < 0.01 compared with DC2.4 expressing mock-GFP (ANOVA, Bonferroni's multiple comparison test). (**e**) DC2.4, DC2.4 expressing mock-GFP, and DC2.4 expressing Clec4A4-GFP were stimulated or not stimulated with LPS for the period indicated, at which time cells were lysed. Total lysate was analyzed using Ab specific for p65 or phosphorylated p65. All data are representative of at least three independent experiments.

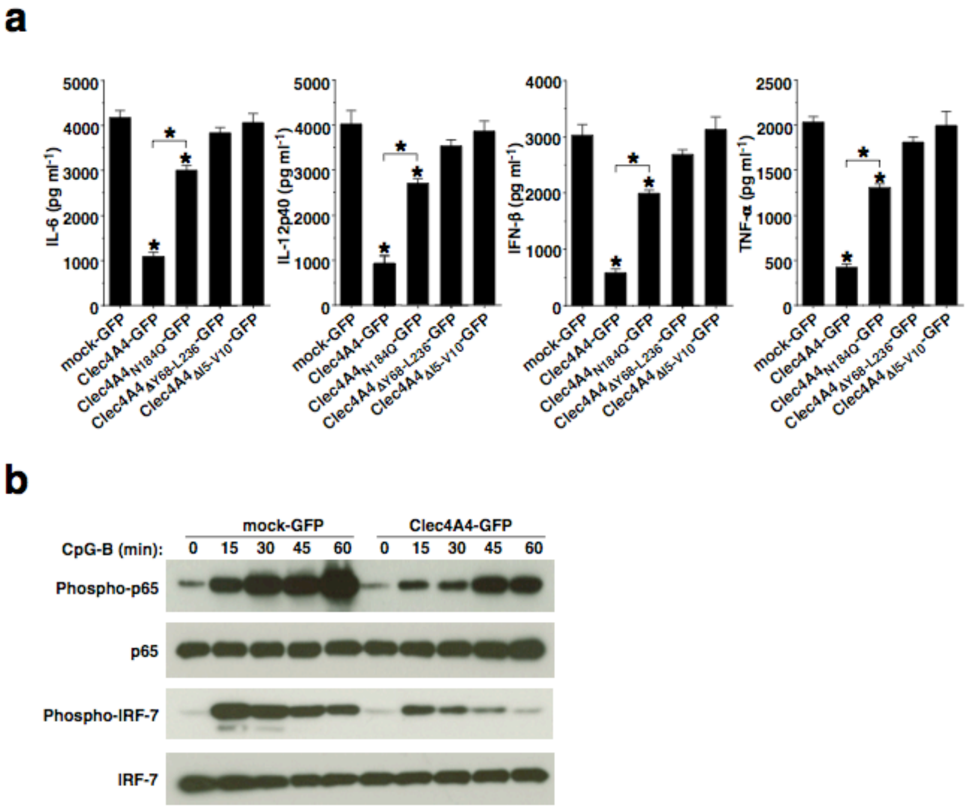

Supplementary Figure 2 Retroviral transduction of Clec4A4 suppresses the TLR-mediated signaling in cDCs. (**a**) BMDCs expressing mock-GFP, Clec4A4-GFP, Clec4A4$_{N186Q}$-GFP, Clec4A4$_{\Delta Y68\text{-}L236}$-GFP, or Clec4A4$_{\Delta I5\text{-}V10}$-GFP were stimulated or not stimulated with the indicated CpG-B, and the production of cytokines was measured by ELISA. Data are the mean ± s.d. from three individual samples in a single experiment. *P < 0.01 compared with BMDCs expressing mock-GFP or among groups (ANOVA, Bonferroni's multiple comparison test). (**b**) BMDCs expressing mock-GFP or Clec4A4-GFP were stimulated or not stimulated with CpG-B for the period indicated, at which time cells were lysed. Total lysate was analyzed using Ab specific for p65 and IRF-7 or for phosphorylated versions of these proteins. All data are representative of at least three independent experiments.

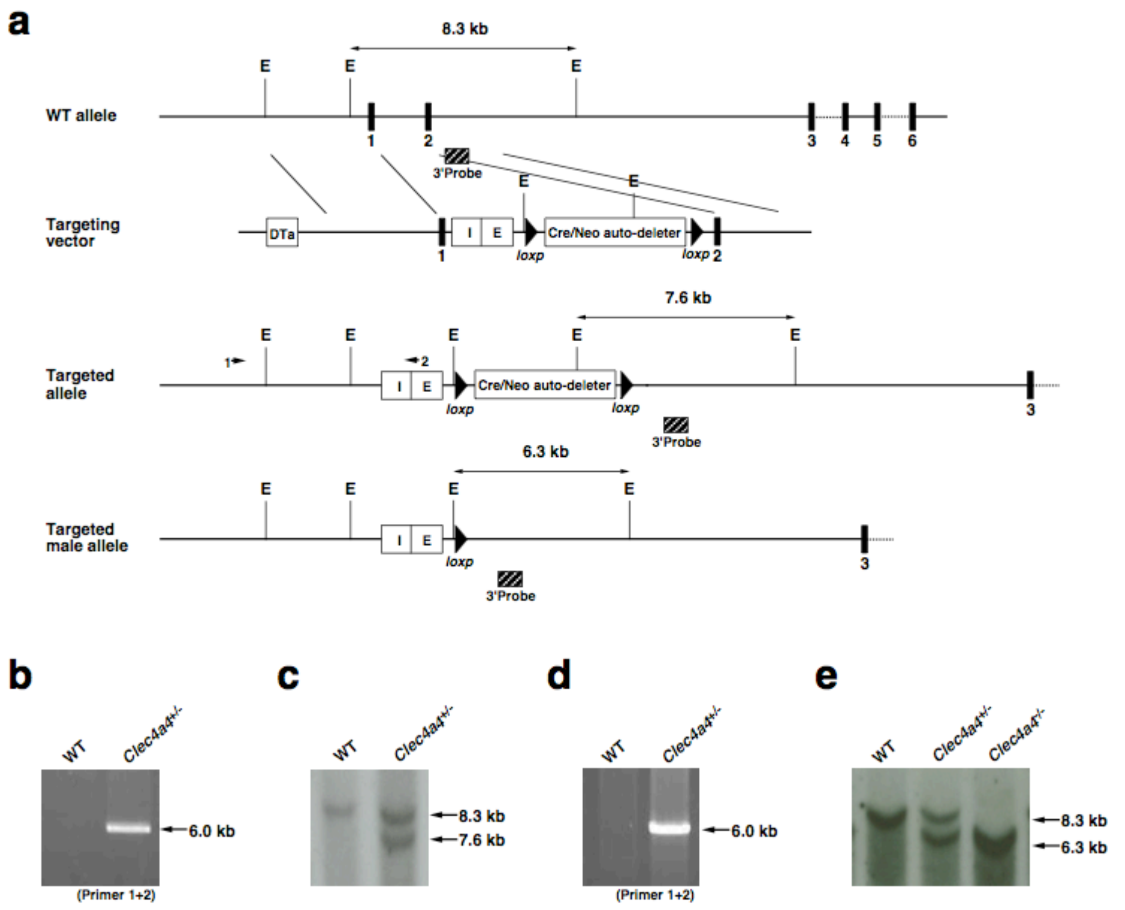

Supplementary Figure 3 Generation and identification of *Clec4a4*$^{-/-}$ mice. (**a**) Strategy used to produce the *Clec4a4*$^{-/-}$ mice. (1) Partial restriction map of the WT *Clec4a4* gene. Exons are depicted as black boxes. The restriction site indicated is E: *Eco*RV (2) Targeting vector used for the introduction of the mutations in the *Clec4a4* gene. A *Sal*I site engineered in place of the start codon in exon 1 of the *Clec4a4* gene was used to clone the IRES-EGFP-Cre/Neo^{r} auto-deleter cassette. DTa: diphtheria toxin a expression cassette, I: IRES, E: EGFP, C/N auto-deleter: Cre/Neo^{r} auto-deleter. The Cre/Neo^{r} auto-deleter cassette is shown bracketed by Lox P sites (filled triangles); it directs its own excision as it passes through the male germline. (3) Structure of the targeted allele following homologous recombination in ESC clones. (4) Structure of the *Clec4a4* allele following expression of the Cre recombinase and excision of the Neo^{r} cassette in mutant mice. The 3' external single-copy probe (a hatched box) and the PCR primers at the 5' end (blackarrows) used to verify proper homologous recombination events are shown. (**b**,**c**) DNA-PCR (**b**) and Southern blot (**c**) analysis of WT and recombinant ESC clones. (**d**,**e**) Genotyping of tail DNA from WT mice and from heterozygous or homozygous mice for the *Clec4a4* allele by DNA-PCR (**d**) and Southern blot (**e**) analysis. In the Southern blot analysis, genomic DNA was digested by *Eco*RV and hybridized to the 3' external single-copy probe. All data are representative of at least three independent experiments.

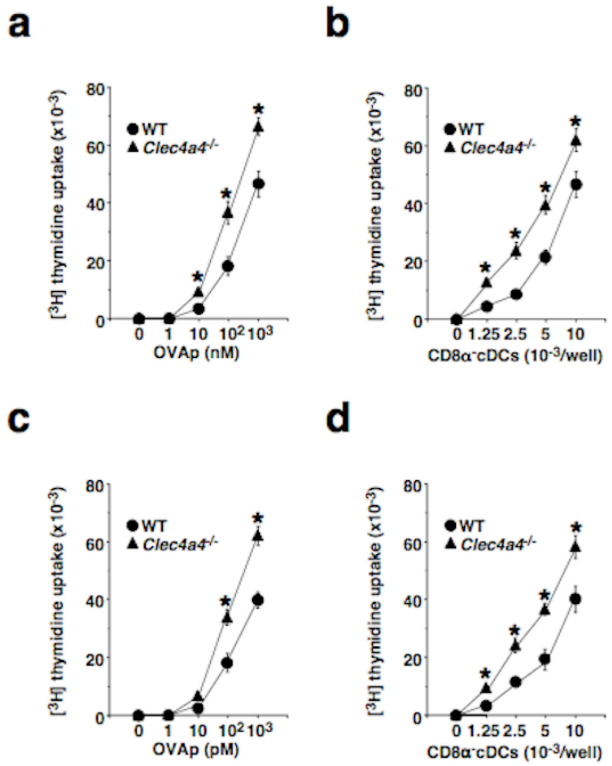

Supplementary Figure 4 Deficiency of Clec4A4 enhances TLR9-mediated activation of CD8α^- cDCs. WT mice (n=6) and *Clec4a4*$^{-/-}$ mice (n=6) were injected with CpG-B, and CD8α^- cDCs were obtained 24 hrs after injection. CD45.1$^+$OT-II CD4$^+$ T cells (**a,b**) or CD45.1$^+$OT-I CD8$^+$ T cells (**c,d**) were cultured with CD8α^- cDCs (1.25x10^3-10^4) obtained from WT mice and *Clec4a4*$^{-/-}$ mice in the presence or absence of $OVA_{323\text{-}339}$ peptide 1 nM-1 μM; **a,b**) or $OVA_{257\text{-}264}$ peptide (1 pM-1 nM; **c,d**), and the proliferation was measured by [^{3}H]thymidine incorporation. Data are the mean ± s.d. from six individual samples in a single experiment. *$P < 0.01$ compared with WT mice (ANOVA, Bonferroni's multiple comparison test). All data are representative of at least three independent experiments.

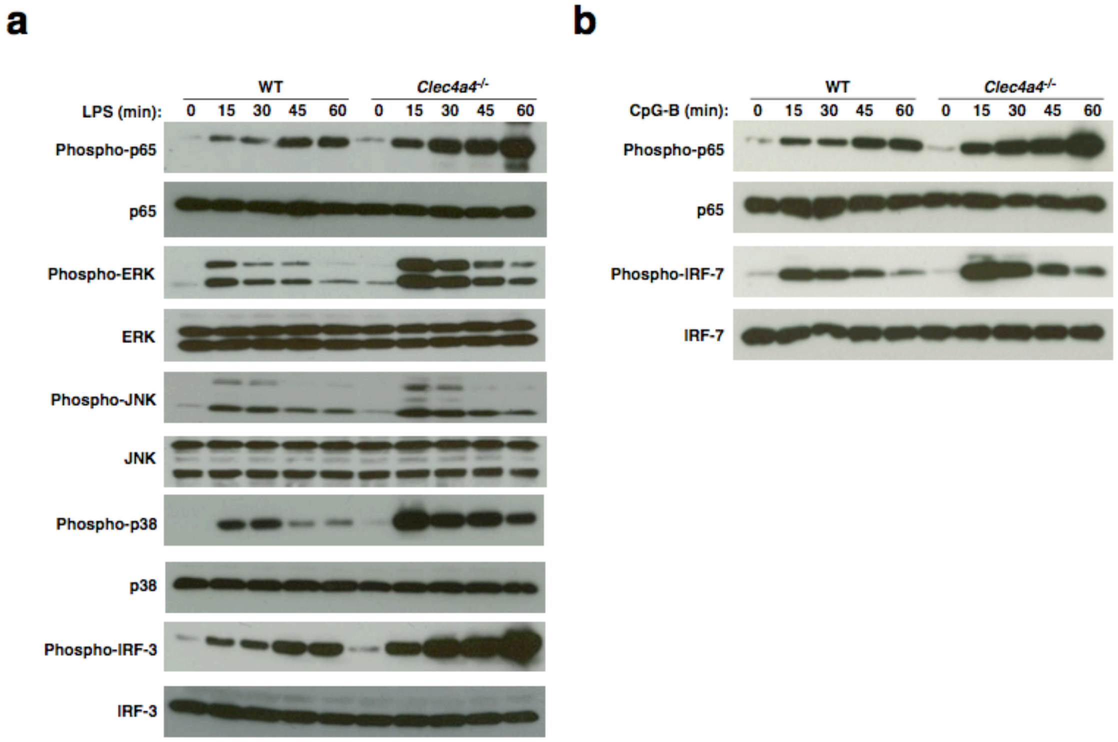

Supplementary Figure 5 Deficiency of Clec4A4 amplifies TLR-mediated signaling in CD8α⁻ cDCs. CD8α⁻ cDCs obtained from WT mice and *Clec4a4*$^{-/-}$ mice were stimulated or not stimulated with LPS (**a**) or CpG-B (**b**) for the period indicated, at which time cells were lysed. Total lysate was analyzed using Ab specific for p65 (**a,b**), ERK (**a**), JNK (**a**), p38 (**a**), IRF-3 (**a**), and IRF-7 (**b**) or for phosphorylated versions of these proteins. All data are representative of at least three independent experiments.

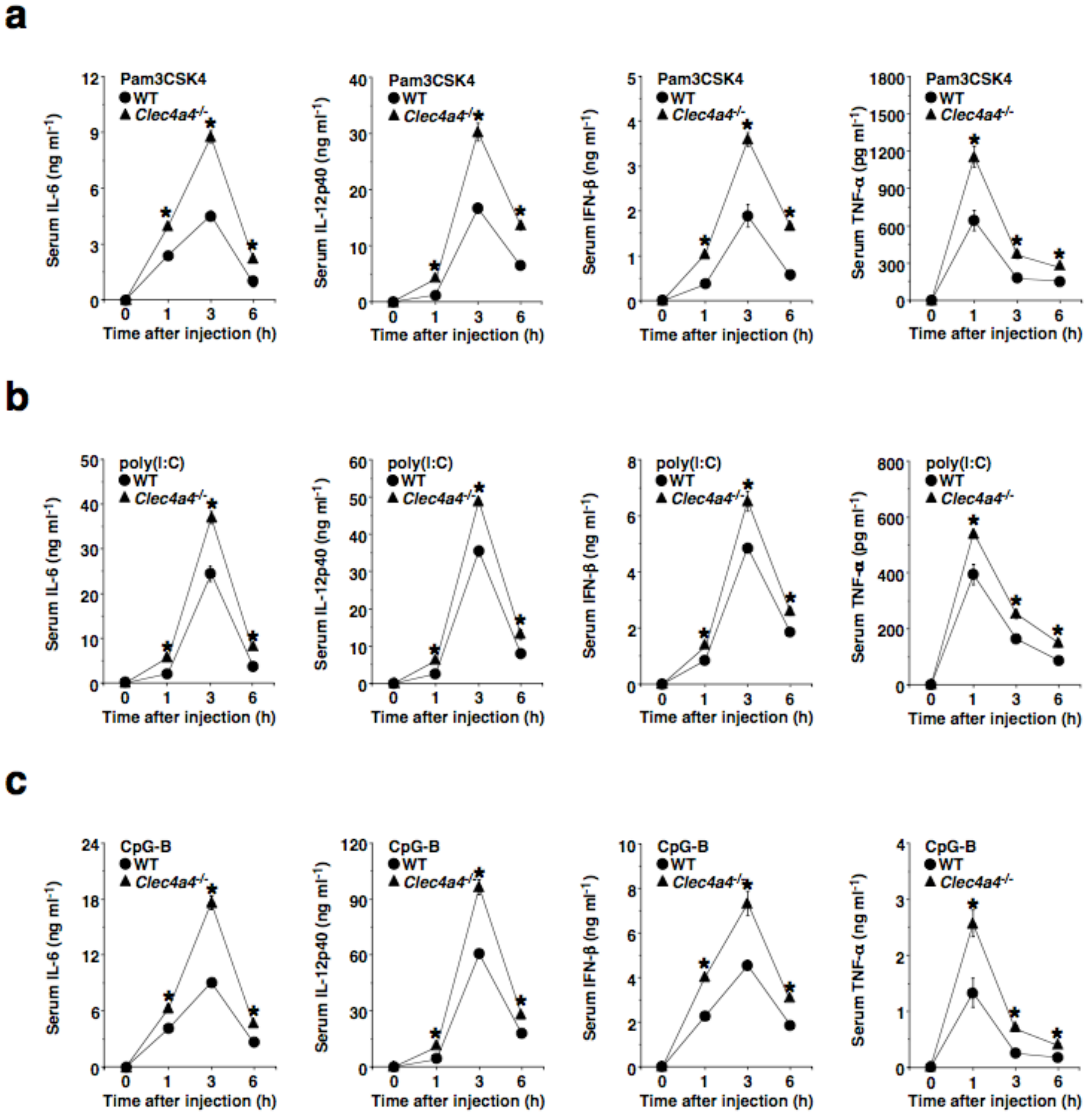

Supplementary Figure 6 Deficiency of Clec4A4 promotes TLR-mediated cytokine production *in vivo*. WT mice (n=6) and *Clec4a4*$^{-/-}$ mice (n=6) were injected with Pam3CSK4 (**a**), poly(I:C) (**b**), and CpG-B (**c**), and serum production of cytokines was measured at the indicated time after injection by ELISA. Data are the mean ± s.d. from six individual samples in a single experiment. *P < 0.01 compared with WT mice (ANOVA, Bonferroni's multiple comparison test). All data are representative of at least three independent experiments.

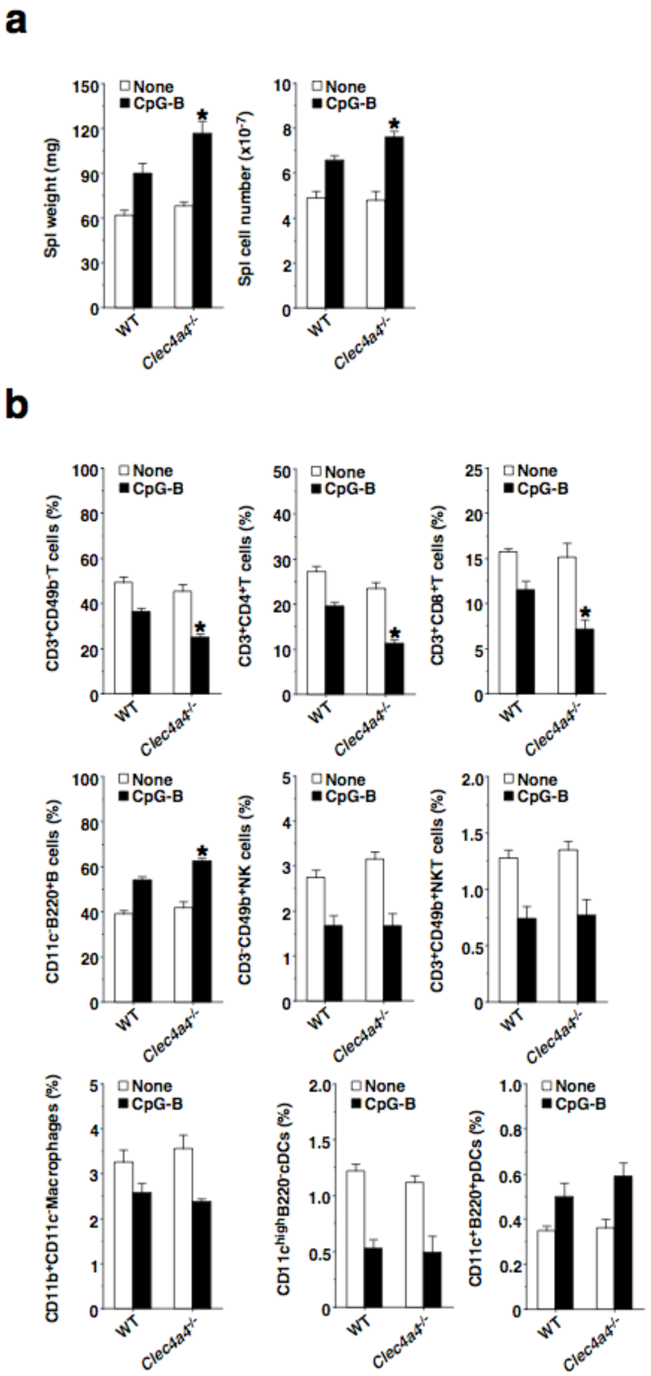

Supplementary Figure 7 Deficiency of Clec4A4 augments inflammatory response *in vivo*. WT mice (n=6) and *Clec4a4*$^{-/-}$ mice (n=6) were injected with or without CpG-B, and Spl were obtained 24 hrs after the injection. The weight of Spl (**a**, left panel), the absolute number of leukocytes (**a**, right panel), and the frequency of the indicated leukocytes (**b**) were analyzed by flow cytometry. Data are the mean ± s.d. from six individual samples in a single experiment. *P < 0.01 compared with WT mice (ANOVA, Bonferroni's multiple comparison test). All data are representative of at least three independent experiments.

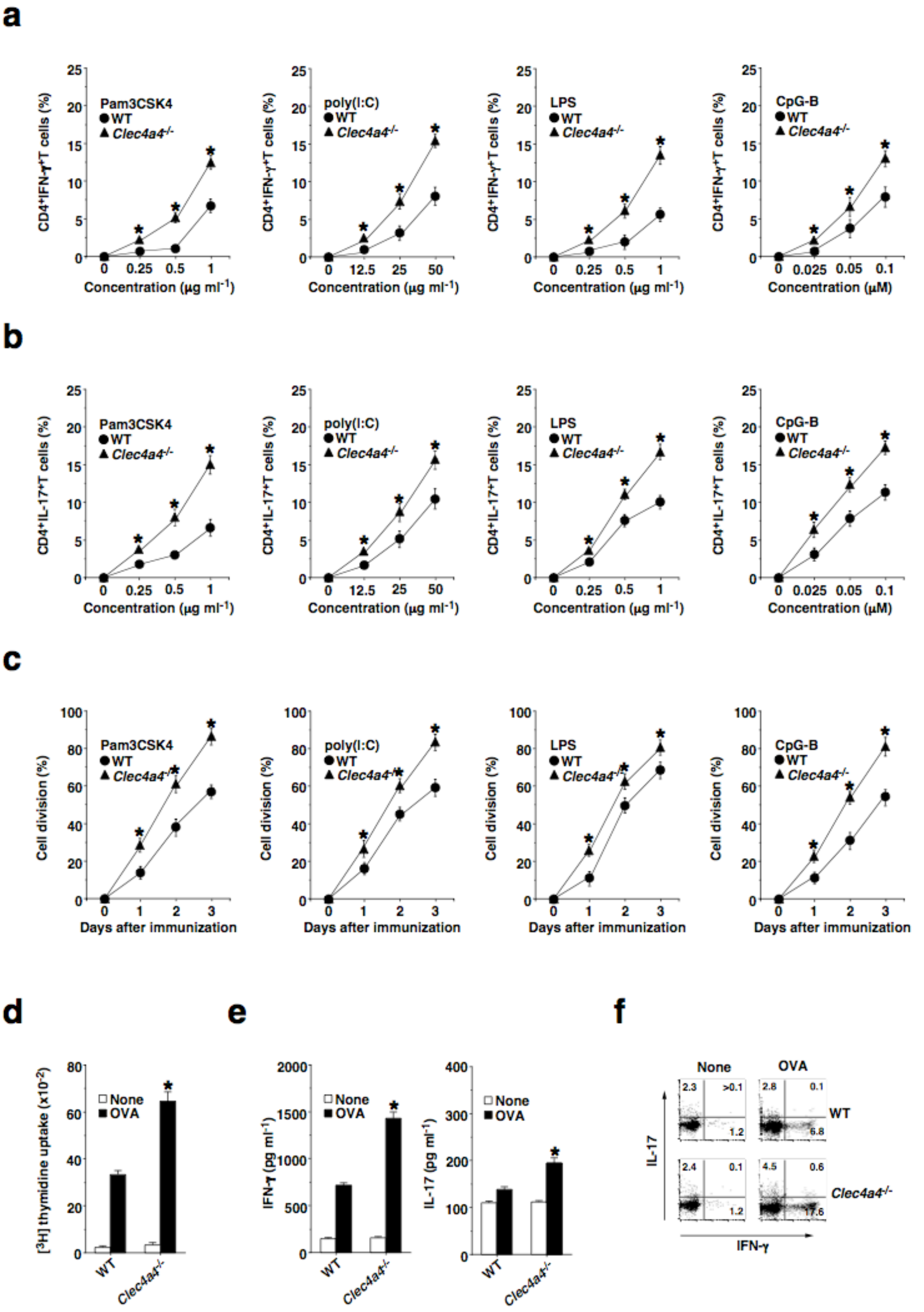

Supplementary Figure 8 Deficiency of Clec4A4 promotes the generation of Ag-specific CD4^{+} T$_{\mathrm{eff}}$ cells. (**a,b**) CD45.1^{+}OT-II CD4^{+} T cells were cultured with CD8α^{-} cDCs obtained from WT mice and *Clec4a4*$^{-/-}$ mice in the presence or absence of

Pam3CSK4 (1.25-1 μg/ml), poly(I:C) (12.5-50 μg/ml), LPS (0.25-1 μg/ml), or CpG-B (0.025-0.1 μM) in combination with $OVA_{323-339}$ peptide under T_H1 (**a**)- or T_H17 (**b**)-polarized culture conditions for 3 days, and intracellular production of IFN-γ (**a**) or IL-17 (**b**) in the cultured $CD4^+$ T cells was analyzed by flow cytometry. Data are the mean percentage of IFN-γ^+ cells or IL-17^+ cells among gated $CD4^+$ T cells ± s.d. from three individual samples in a single experiment. (**c**) CFSE-labeled $CD45.1^+$OT-II $CD4^+$ T cells were transferred into WT mice (n=6) and *Clec4a4*$^{-/-}$ mice (n=6), and then the mice were immunized with OVA protein in combination with or without the indicated TLR ligands. Ag-specific division of $CD45.1^+$OT-II $CD4^+$ T cells was analyzed at indicated days after the immunization by flow cytometry. Data are the mean percentage of the dividing cells ± s.d. from six individual samples in a single experiment. (**d-f**) WT mice (n=6) and *Clec4a4*$^{-/-}$ mice (n=6) were immunized with CpG-B plus OVA protein. At 14 days after the immunization, Spl $CD4^+$ T cells were isolated then cultured with WT $CD11c^+$ DCs in the presence or absence of OVA protein for the measurement of proliferative responses by [^{3}H]thymidine incorporation (**d**), and production of IFN-γ (**e**, left panel) and IL-17 (**e**, right panel) by ELISA. Data are the mean ± s.d. from six individual samples in a single experiment. (**f**) Intracellular production of IFN-γ and IL-17 in the cultured $CD4^+$ T cells was analyzed by flow cytometry. Data are presented by a dot plot, and numbers represent the proportion of IFN-γ^+ cells and IL-17^+ cells among gated $CD4^+$ T cells in each quadrant. $^*P < 0.01$ compared with WT mice (ANOVA, Bonferroni's multiple comparison test). All data are representative of at least three independent experiments.

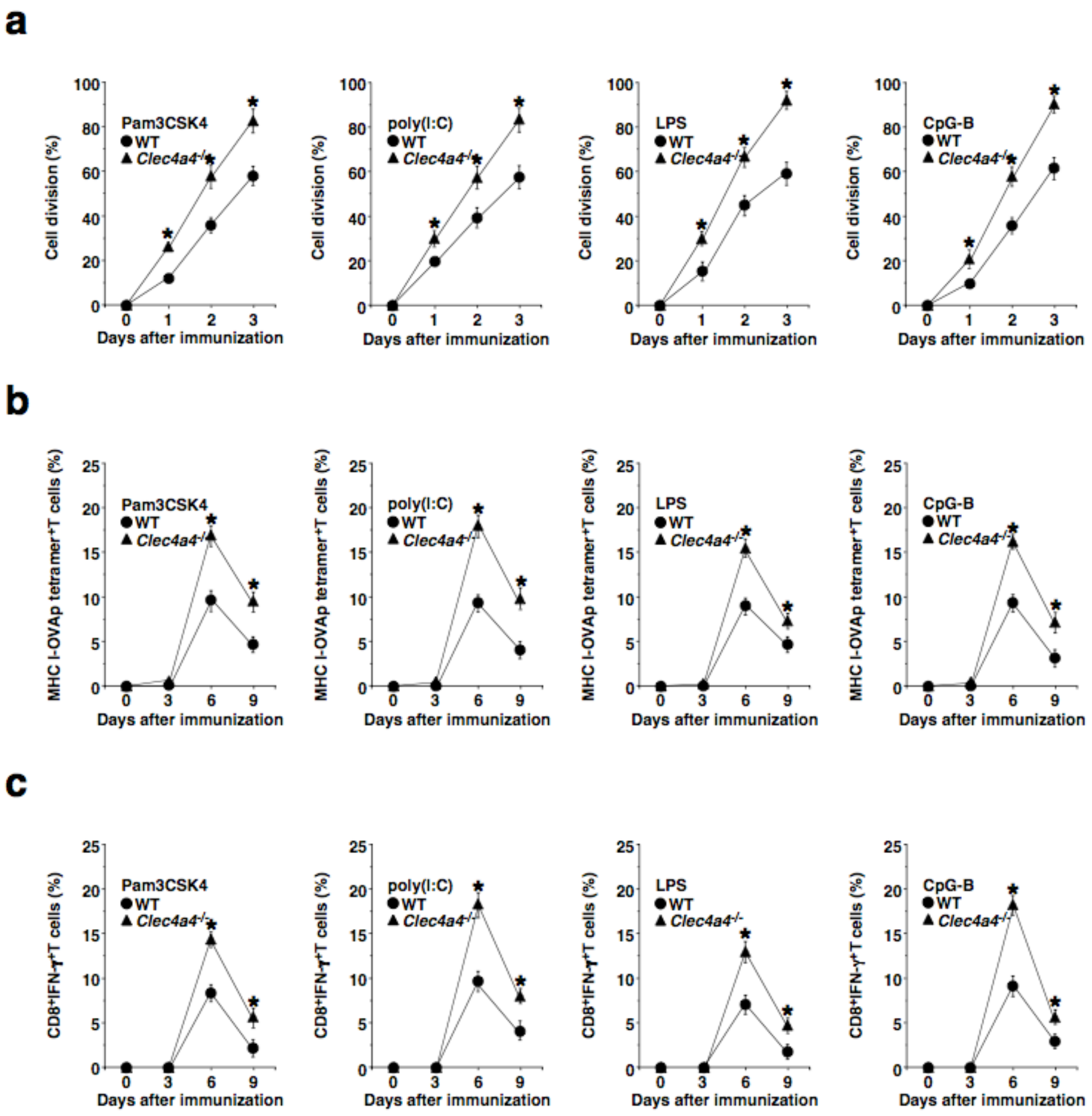

Supplementary Figure 9 Deficiency of Clec4A4 promotes the generation of Ag-specific CD8$^+$ T_{eff} cells. (**a**) CFSE-labeled CD45.1$^+$OT-I CD8$^+$ T cells were transferred into WT mice (n=6) and *Clec4a4*$^{-/-}$ mice (n=6), and then the mice were immunized with OVA protein in combination with or without the indicated TLR ligands. Ag-specific division of CD45.1$^+$OT-I CD8$^+$ T cells was analyzed at indicated days after the immunization by flow cytometry. Data are the mean percentage of the dividing cells ± s.d. from six individual samples in a single experiment. (**b,c**) WT mice (n=6) and *Clec4a4*$^{-/-}$ mice (n=6) were immunized with the indicated TLR ligands, anti-CD40 mAb, and OVA protein. At 3-9 days after the immunization, splenocytes were analyzed for the generation of MHC I-OVA tetramer$^+$CD44^{high}CD8$^+$ T cells (**b**), and for intracellular IFN-γ-producing CD8$^+$ T cells (**c**) by flow cytometry. Data are the mean percentage of MHC I-OVA tetramer$^+$CD44^{high} cells (**b**) or IFN-γ$^+$ cells (**c**) among gated CD8$^+$ T cells ± s.d. from six individual samples in a single experiment. *P < 0.01 compared with WT mice (ANOVA, Bonferroni's multiple comparison test). All data are representative of at least three independent experiments.

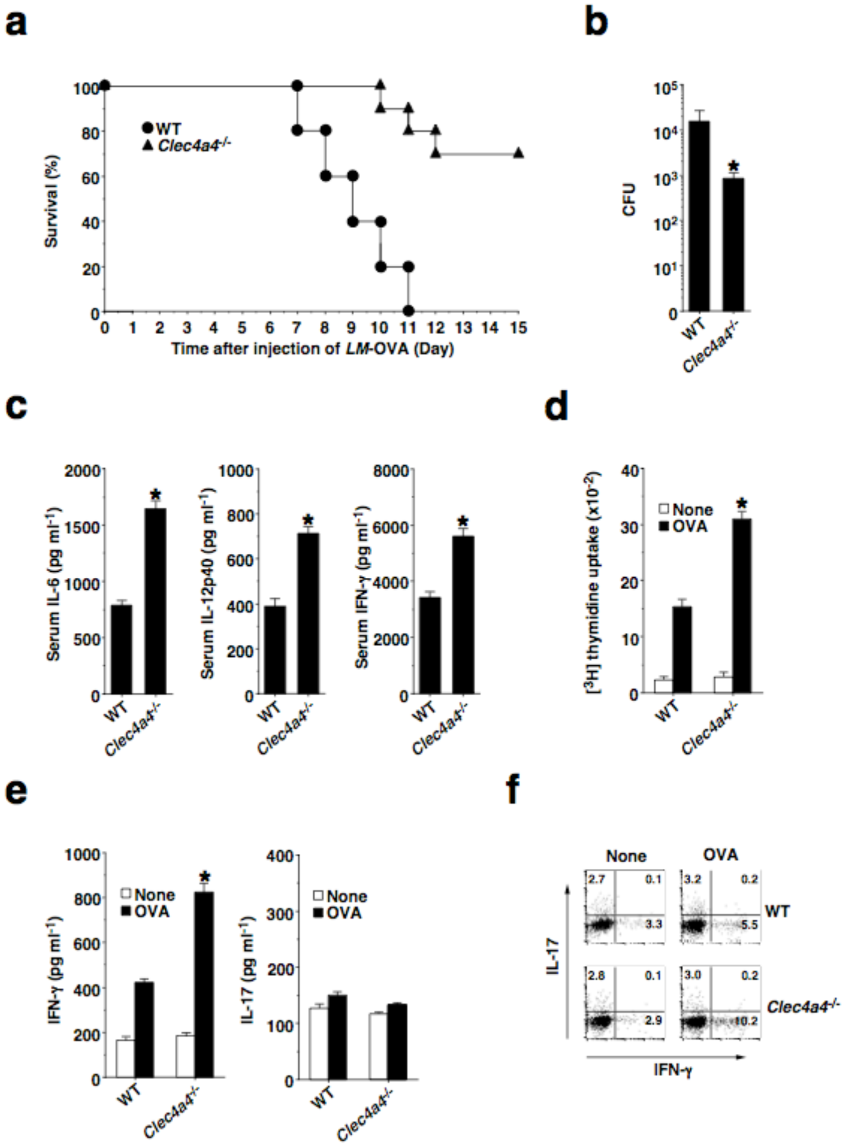

Supplementary Figure 10 Deficiency of Clec4A4 promotes host protective immune responses against bacterial infection. (**a-c**) WT mice (n=10) and *Clec4a4*$^{-/-}$ mice (n=10) were infected with *LM*-OVA. (**a**) Survival rate was monitored at the indicated times for 15 days after infection with *LM*-OVA. *P < 0.01 compared with WT mice (Kaplan-Meier log-rank test). (**b**) Bacterial burden in the Spl was determined as colony forming unit (CFU) 6 days after infection with *LM*-OVA. (**c**) Serum production of cytokines was measured 6 days after infection with *LM*-OVA by ELISA. Data are the mean ± s.d. from ten individual samples in a single experiment. *P < 0.01 compared with WT mice (ANOVA, Bonferroni's multiple comparison test). (**d-f**) WT mice (n=6) and *Clec4a4*$^{-/-}$ mice (n=6) were infected with *LM*-OVA. At 6 days after infection with *LM*-OVA, Spl CD4^{+} T cells were cultured with WT CD11c^{+} DCs in the presence or absence of OVA protein for the measurement of proliferative responses by [^{3}H]thymidine incorporation (**d**), and production of IFN-γ (**e**, left panel) and IL-17 (**e**, right panel) by ELISA. Data are the mean ± s.d. from six individual samples in a single experiment. *P < 0.01 compared with WT mice (ANOVA, Bonferroni's multiple

comparison test). (**f**) Intracellular production of IFN-γ and IL-17 in the cultured $CD4^+$ T cells was analyzed by flow cytometry. Data are presented by a dot plot, and numbers represent the proportion of IFN-γ^+ cells and IL-17^+ cells among gated $CD4^+$ T cells in each quadrant. All data are representative of at least three independent experiments.

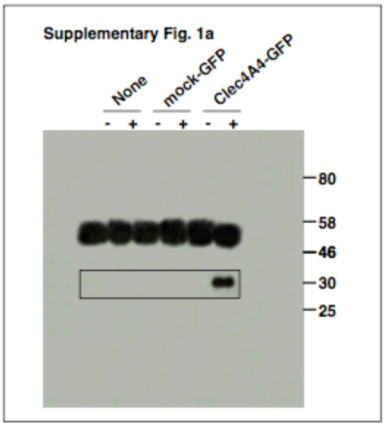

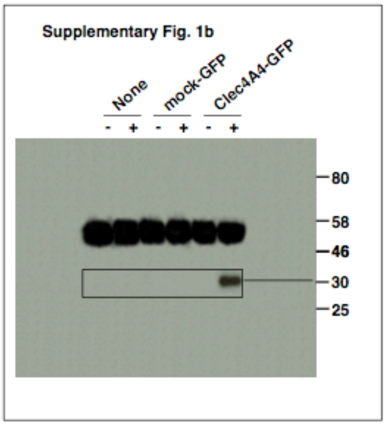

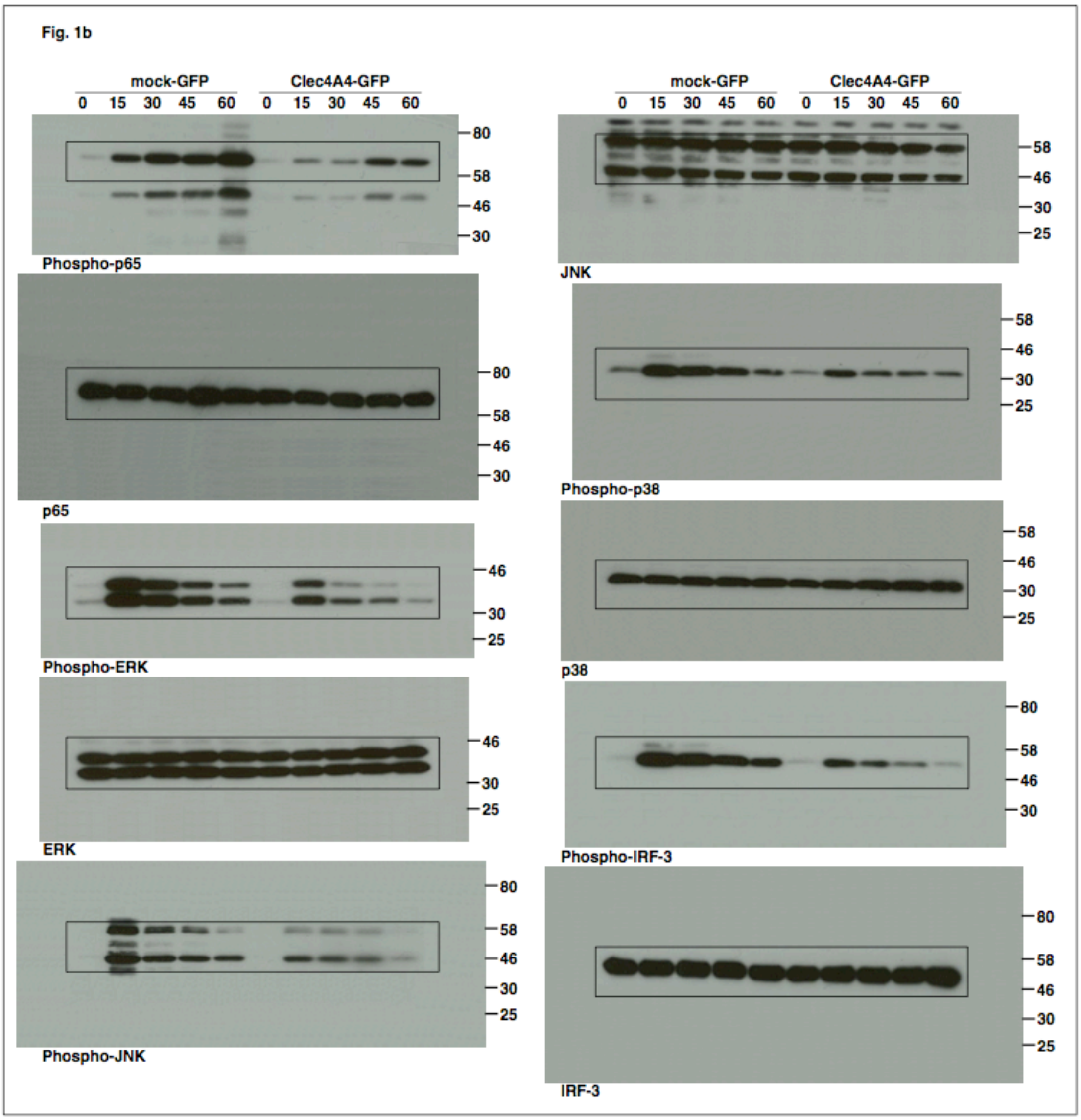

Supplementary Figure 11 Full blots of Supplementary Fig. 1a, Supplementary Fig. 1b, and Fig. 1b.

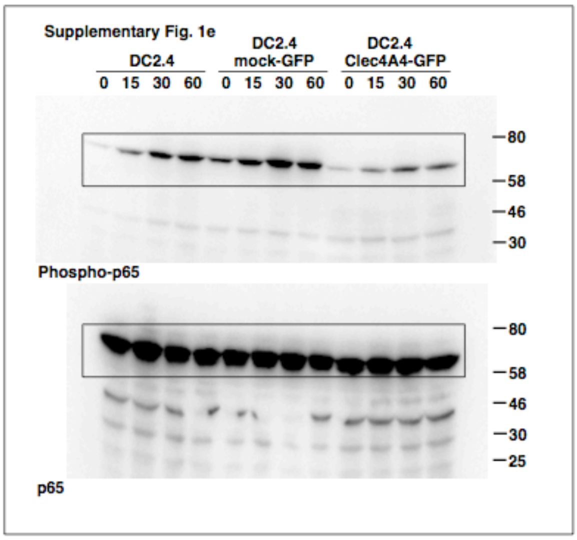

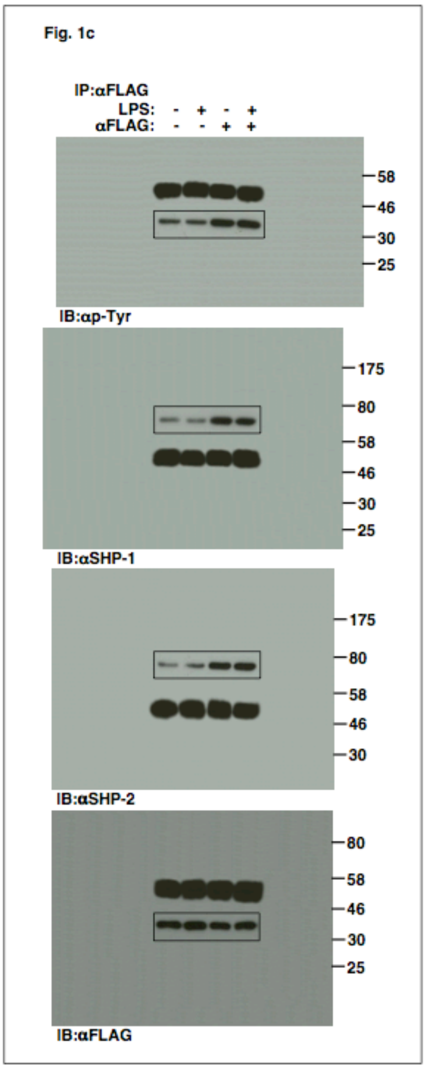

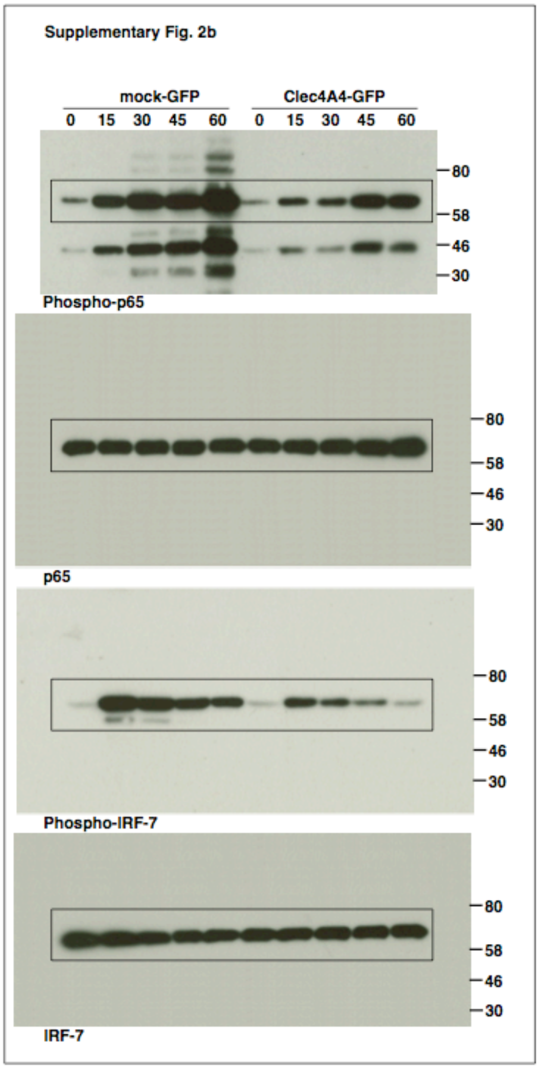

Supplementary Figure 12 Full blots of Supplementary Fig. 1e, Supplementary Fig. 2b, and Fig. 1c.

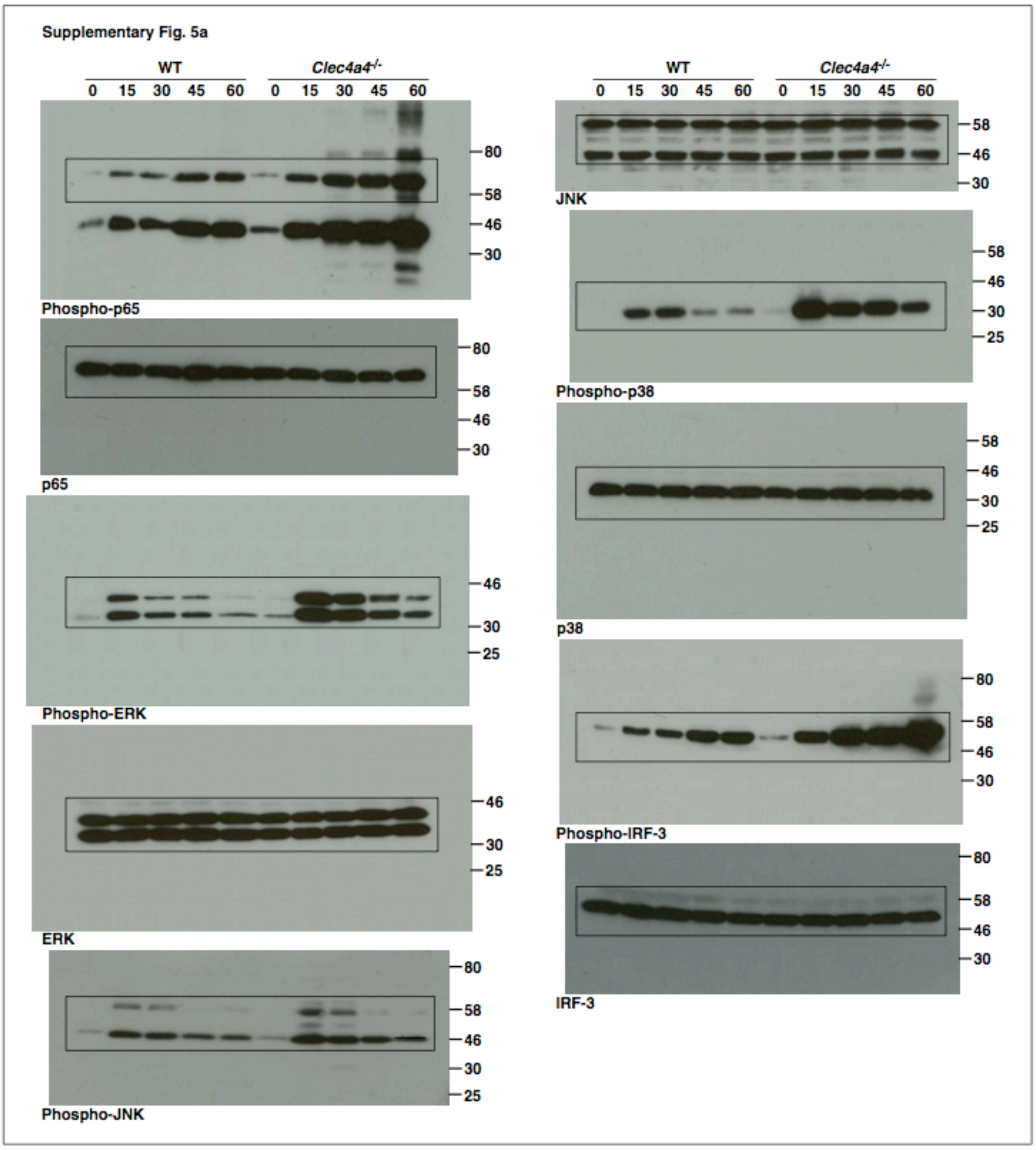

Supplementary Figure 13 Full blots of Supplementary Fig. 5a.

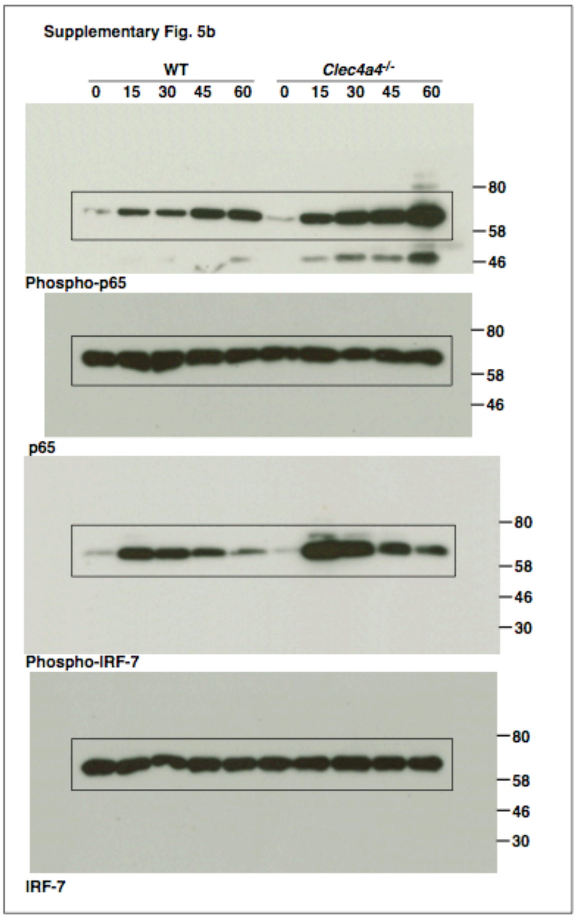

Supplementary Figure 14 Full blots of Supplementary Fig. 5b.
